# Single-cell RNA sequencing in donor and end-stage heart failure patients identifies NLRP3 as a therapeutic target for arrhythmogenic right ventricular cardiomyopathy

**DOI:** 10.1186/s12916-023-03232-8

**Published:** 2024-01-08

**Authors:** Mengxia Fu, Xiumeng Hua, Songren Shu, Xinjie Xu, Hang Zhang, Zhiming Peng, Han Mo, Yanyun Liu, Xiao Chen, Yicheng Yang, Ningning Zhang, Xiaohu Wang, Zirui Liu, Guangxin Yue, Shengshou Hu, Jiangping Song

**Affiliations:** 1 Beijing Key Laboratory of Preclinical Research and Evaluation for Cardiovascular Implant Materials, Animal Experimental Centre, Fuwai Hospital, National Center for Cardiovascular Disease, Chinese Academy of Medical Sciences and Peking Union Medical College, Beijing, 100037 China; 2 Galactophore Department, Galactophore Center, Beijing Shijitan Hospital, Capital Medical University, Beijing, China; 3 State Key Laboratory of Cardiovascular Disease, Fuwai Hospital, National Center for Cardiovascular Diseases, Chinese Academy of Medical Sciences and Peking Union Medical College, 167A Beilishi Road, Xi Cheng District, Beijing, 10037 China; 4 Department of Orthopedics, Peking Union Medical College Hospital, Chinese Academy of Medical Sciences and Peking Union Medical College, Beijing, China; 5 Shenzhen Key Laboratory of Cardiovascular Disease, Fuwai Hospital Chinese Academy of Medical Sciences, Shenzhen, 518057 China; 6 Engineering Research Center of Molecular and Neuro Imaging of Ministry of Education, School of Life Science and Technology, Xidian University, Shaanxi, 710126 China; 7 Department of Cardiovascular Surgery, Fuwai Hospital, National Center for Cardiovascular Diseases, Chinese Academy of Medical Sciences and Peking Union Medical College, Beijing, 10037 China; 8 The Cardiomyopathy Research Group, Fuwai Hospital, National Center for Cardiovascular Diseases, Chinese Academy of Medical Sciences and Peking Union Medical College, Beijing, 10037 China

**Keywords:** Arrhythmogenic right ventricular cardiomyopathy, Single-cell RNA sequencing, NLRP3

## Abstract

**Background:**

Dilation may be the first right ventricular change and accelerates the progression of threatening ventricular tachyarrhythmias and heart failure for patients with arrhythmogenic right ventricular cardiomyopathy (ARVC), but the treatment for right ventricular dilation remains limited.

**Methods:**

Single-cell RNA sequencing (scRNA-seq) of blood and biventricular myocardium from 8 study participants was performed, including 6 end-stage heart failure patients with ARVC and 2 normal controls. ScRNA-seq data was then deeply analyzed, including cluster annotation, cellular proportion calculation, and characterization of cellular developmental trajectories and interactions. An integrative analysis of our single-cell data and published genome-wide association study-based data provided insights into the cell-specific contributions to the cardiac arrhythmia phenotype of ARVC. Desmoglein 2 (Dsg2)^mut/mut^ mice were used as the ARVC model to verify the therapeutic effects of pharmacological intervention on identified cellular cluster.

**Results:**

Right ventricle of ARVC was enriched of *CCL3*^+^ proinflammatory macrophages and *TNMD*^+^ fibroblasts. Fibroblasts were preferentially affected in ARVC and perturbations associated with ARVC overlap with those reside in genetic variants associated with cardiac arrhythmia. Proinflammatory macrophages strongly interact with fibroblast. Pharmacological inhibition of Nod-like receptor protein 3 (NLRP3), a transcriptional factor predominantly expressed by the *CCL3*^+^ proinflammatory macrophages and several other myeloid subclusters, could significantly alleviate right ventricular dilation and dysfunction in Dsg2^mut/mut^ mice (an ARVC mouse model).

**Conclusions:**

This study provided a comprehensive analysis of the lineage-specific changes in the blood and myocardium from ARVC patients at a single-cell resolution. Pharmacological inhibition of NLRP3 could prevent right ventricular dilation and dysfunction of mice with ARVC.

**Supplementary Information:**

The online version contains supplementary material available at 10.1186/s12916-023-03232-8.

## Background

Arrhythmogenic right ventricular cardiomyopathy (ARVC) is a rare inherited heart disease, mainly involving the right ventricle. Importantly, dilation without visible affection of ventricular function may be the first right ventricular changes, which may imply that the patient is transitioning to a level of higher risk of ventricular arrhythmia and heart failure [1, 2]. Currently, the medical treatment for right ventricle dilation of ARVC is still dissatisfactory, partially due to the knowledge gap between the pathological remodeling and underlining pathogenesis.

Recent studies partially showed the pathogenesis of pathological remodeling and subsequent structural/functional alterations for ARVC at the cellular level [3–5]. For example, inflammatory cells were observed in the heart of ARVC patients [6]. In the Dsg2 mutant mice, T cells and macrophages were enriched in the heart [3]. A study identified a mix of macrophages, neutrophils, and mast cells in the right ventricle of ARVC patients [4]. However, most related studies are limited on traditional definition of cell types but neglect the complicated cell heterogeneity at a higher resolution, which might miss valuable information like pathogenic cellular subpopulations. Therefore, it is essential to comprehensively map the cell atlas of human ARVC, which is the fundamental of medical treatment development.

In this study, we performed single-cell RNA sequencing (scRNA-seq) of non-cardiomyocytes (NCMs) in bi-ventricle and peripheral blood mononuclear cells (PBMCs) in blood from ARVC patients and normal control (NC) to dissect the heterogeneity and functionality at the transcriptomic level. We revealed different cellular landscape between NC and ARVC, as well as between the bi-ventricle of ARVC. Nod-like receptor protein 3 (NLRP3) inhibition might be a potential therapy to alleviate right ventricular dilation and dysfunction of ARVC.

## Methods

### Human specimen collection

This study was approved by the Ethics Committee of Fuwai Hospital, Chinese Academy of Medical Sciences and Peking Union Medical University, as well as by the Ministry of Science and Technology, with the approval number of FW-2022–0043. Written informed consent for tissue donation, which clearly stated the purpose of our study, was obtained from all of the patients. The diagnosis of ARVC was confirmed via a comprehensive assessment of clinical, magnetic resonance imaging, and pathological results as we have previously described [7] (Additional file 1: Table S1). The pathological result of myocardium was the gold standard for diagnosing ARVC, and the pathological pictures were assessed by two pathologists at Fuwai Hospital. Blood, left and right ventricle myocardium samples were obtained from ARVC patients and NC. Diseased hearts were obtained from 6 ARVC patients with end-stage heart failure undergoing heart transplantation (HTx). The ARVC patients did not receive ventricular assist device or chronic inotropic therapy prior heart transplant. Two NC hearts were obtained from brain-death donors with normal circulatory supply, who were not suitable for transplantation due to the technical or non-cardiac reasons by following the guideline of China Transplant Services. A table with the clinical characteristics of the patients and normal controls was provided in Additional file 1: Table S2.

### Isolation of cardiac cells

Myocardium was collected in 10 mL Dulbecco’s modified Eagle’s medium (DMEM; 11,885,084, Thermo Fisher, Waltham, MA) containing 10% fetal bovine serum (FBS; 10,091,148, Thermo Fisher, Waltham, MA) on ice. The epicardium and endocardium were removed. Samples were washed by phosphate buffered saline (PBS) to remove the remaining blood cells and then cut into 1-mm^3^ pieces and digested in Hanks’ Balanced Salt Solution (HBSS; 14175095, Thermo Fisher, Waltham, MA) containing 600 U/mL Collagenase, type 2 (LS004176, Worthington Biochemical, Lakewood, CA) at 37 °C for 15 min with gently shaking. The supernatant was filtered with a 40-μm cell strainer (431750, Corning, NY, USA) and mixed with equal volume of 10% FBS/DMEM collected by centrifugation at 300* g*, 4 °C for 5 min. The supernatant was discarded, and the cell pellets were resuspended in 1 mL of 2% FBS/DMEM on ice. These steps were repeated 3 times. Next, the cell pellet was resuspended in 1 ml red blood cell lysis buffer (C3702, Beyotime, Shanghai, China) and incubated for 5 min at room temperature. The resulting suspension was further diluted to 11 ml with PBS, then filtered and centrifuged at 300* g*, 4 °C for 5 min. The cell precipitate was washed twice with PBS containing 2% FBS (washing buffer, WB) and finally resuspended in 500 µl WB on ice. In addition, peripheral blood was collected into EDTA-containing tubes, and the mononuclear cells were isolated by gradient centrifugation (HISTOPAQUE-1077, Sigma–Aldrich). The cell pellets were resuspended in WB.

### Flow cytometry and cell sorting

The single-cell suspensions consisting of immune cells and non-immune cells were stained with BB515 mouse anti-human CD45 (564585, BD, CA, USA) at a concentration of 1:200 on ice in the dark for 30 min, washed with WB and centrifuged at 300* g*, 4 °C for 5 min. The washing step was repeated twice. Then the cells were resuspended with 500 µl WB and stained with 7-AAD (559925, BD, CA, USA) 1:20 and analyzed on a FACSAria II cell sorter (FACSAria II, BD biosciences, CA, USA). Live non-immune cells (7-AAD negative/CD45 positive cells) were sorted for scRNA-seq.

### Single-cell library construction and sequencing

Single-cell suspensions were processed through the 10 × Genomics Chromium System (10 × Genomics, Pleasanton, CA, USA), followed by the construction of 3’ gene expression v3.1 libraries and sequencing on an Illumina Noveseq6000 sequencer. Briefly, cells (> 90% viability), reagents, gel beads, and partitioning oil are loaded onto 10 × Chromium Chip B. Ideally, each individual cell is wrapped with an oil drop that contains gel beads and reagents. This creates an independent reaction space for each cell called gel bead in emulsion (GEM). The primers provided by the gel bead contain a sequencing primer, barcode, UMI, and poly (dT) sequence. The cell in the GEM is lysed, and incubation of the GEM produces barcoded, full-length cDNA from poly-adenylated mRNA. Next, all cDNAs are pooled together, and the steps of general library construction are performed including amplification, fragmentation, end repair, adapter ligation, and sample index polymerase chain reaction (PCR) (98 °C for 45 s; [98 °C for 20 s, 67 °C for 30 s, and 72 °C for 1 min] × 14 cycles; 72 °C for 1 min). The libraries were then sequenced using Illumina Noveseq6000 by CapitalBio Technology (Beijing, China). Sequencing data are available at Genome Sequence Archive (accession no. HRA005871).

### scRNA‑seq data preprocessing

The scRNA-seq data in FASTA files was processed by Cell Ranger software (Version 6.1.2, 10 × genomics). The sequencing reads were demultiplexed, mapped to the GRCh38 human reference genome, and counted by unique molecular identifier (UMI). Then the UMI count matrix was analyzed using the Seurat package (v3.2.0) in R software (v3.6.1). Cells expressing hemoglobin genes were removed from the data set, as they likely represented erythrocytes. Only the cells with 200 ~ 4000 detected genes and < 10% mitochondrial UMIs were considered valid cells and were then used for downstream analysis [8]. The counts of different biotypes were summarized in Additional file 1: Table S3.

### Dimensional reduction and clustering

The UMI counts were next normalized for library size and log-transformed using Seurat’s default normalization parameters (normalization.method = "LogNormalize", scale.factor = 10,000). Highly variable genes were identified using Seurat and used to perform principal component analysis. To prevent clusters from being biased by mitochondrial transcript content, the gene expression values were scaled based on the cell mitochondrial transcript content. The first 14 principal components were used as an input for SNN clustering and for embedding using the uniform manifold approximation and projection (UMAP). The batch effect among the different samples was corrected using the Seurat V4 integration algorithm. The clustering analysis yielded 37 clusters (C0–C36) (Additional file 2: Fig. S1). We combined SingleR with manual annotation to assign cell types to the different clusters. C32, C33, C35, and C36 were excluded because they contained doublets (C32 and C35) or low-quality cells (characterize low number of features and unclear marker gene expression; C33 and C36) (Additional file 2: Fig. S2; Additional file 1: Table S4 [9–16]); thus, 247,684 cells were included for further analysis.

### Hierarchical clustering of major cellular clusters

To measure the similarities among major cellular clusters in different samples, we calculated the distance between cellular clusters. The distance defined as (1-Pearson correlation coefficient)/2 [17], and the Pearson correlation coefficient was calculated using top 25% highly variable genes.

### Functional enrichment

To identify the potential biological functions of cell clusters, we performed enrichment analysis with marker genes for cell clusters. Marker genes detected in > 25% cells in a cluster with average log-fold-change > 0.5 were used in the analysis. Enrichment analyses were performed using R package clusterProfiler (Version 3.14.3). Over-representation tests on Gene Ontology (GO) Biological Processes terms were performed using clusterProfiler function enrichGO, and comparisons among different cell clusters were performed using clusterProfiler function compareCluster.

Enrichment scores were calculated using Seurat function AddModuleScore. AddModuleScore function calculated the average expression of a gene set subtracting the aggregated expression of control gene sets, which could be deemed as the average relative expression. Genes associated with each term were retrieved from R package org.Hs.eg.db (Version 3.10.0) according to the GO identity of the term.

### Transcription factor activity analysis

To infer potential regulatory transcription factors (TFs) and their target genes in cell clusters, we ran pySCENIC (Version 0.10.3) on the UMI count matrices of major cell populations. In brief, pySCENIC infers TFs and their target genes from correlations between the expression of genes across cells. A TF and its target genes are defined as a regulon. The regulons are then refined by pruning targets based on enriched motifs. Finally, the activity of a regulon is measured by an AUCell value in each single cell. A high AUCell value indicates high activity and enrichment of a regulon in a cell.

### Cell cycle analysis

To predict the cell cycle phases of each individual cells in the given cluster, we used Seurat function CellCycleScoring. The previous well-defined marker genes of S and G2/M phases were used to calculate the S-scores and G2/M-scores, respectively [18].

### Trajectory analysis

Slingshot was used to perform trajectory analysis [19]. In brief, slingshot first identifies the global lineage structure with a cluster-based minimum spanning tree and then fits simultaneous principal curves to describe each lineage and determines the pseudotime of each cell.

### Intercellular communication analysis

To study the intercellular communication among cell populations, R package "CellChat" (version 1.1.3) was applied [20]. Using the “aggregateNet” function in CellChat, the aggregated cell–cell communication network was calculated. The results were visualized using the function "netVisual_circle" and “netVisual_chord_gene”.

### Genetics analysis

The genomic DNA was extracted from peripheral blood according to the manufacturer’s instruction (Blood DNA Extraction Kit, Enriching Biotechnology, China). Probands were screened with 14 ARVC related genes including 5 desmosome genes and nondesmosomal genes including *TMEM43*, *PLN*, *CTNNA3*, *LMNA*, *DES*, transforming growth factor beta 3 (*TGFB3*), Sodium Voltage-Gated Channel Alpha Subunit 5 (*SCN5A*), Titin (*TTN*), and Ryanodine receptor 2 (*RYR2*) by captured next-generation sequencing using Illumina 2500 platform (Illumina, USA). All variants were annotated by the following strategies: (1) variants with a MAF less than 5% in 1000 genomic data (1000g_all) [21], esp6500siv2_all (http://evs.gs.washington.edu/EVS), and gnomAD data (gnomAD_ALL and gnomAD_EAS) [22]. (2) Only single-nucleotide variants (SNVs) occurring in exons or splice sites (splicing junction 10 bp) are further analyzed since we are interested in amino acid changes. (3) Then synonymous SNVs which are not relevant to the amino acid alternation predicted by dbscSNV are discarded. The small fragment non-frameshift (< 10 bp) indel in the repeat region defined by RepeatMasker are discarded. (4) Variations are screened according to scores of SIFT [23], Polyphen [24], MutationTaster [25], and CADD [26] software. The potentially deleterious variations are reserved if the score of more than half of these four software support harmfulness of variations [27]. Sites (> 2 bp) did not affect alternative splicing were removed. (5) For TTN variants, only truncated variants remained for pathogenesis analysis. Sanger sequencing was used to validate putatively pathogenic variants and screen family members.

In order to better predict the harmfulness of variation, the classification system of the American College of Medical Genetics and Genomics was used. The variations are classified into pathogenic, likely pathogenic, uncertain significance, likely benign, and benign [28].

### Immunofluorescence staining

Immunofluorescence staining was performed as previously described [29]. Formaldehyde-fixed paraffin-embedded sections were dewaxed with methanol, subjected to antigen retrieval, blocked for 1 h, and incubated with primary antibodies overnight at 4 °C. After washing with PBS (10,010,023, Thermo Fisher, Waltham, MA) 3 times, the slides were incubated with appropriate fluorescence-labeled secondary antibodies for 30 min at 37 °C. The slides, shielded from light, were washed 3 times in PBS, and then counterstained and mounted with DAPI (ZLI-9557, ZSGB-BIO, Beijing, China). The primary antibodies used were as follows: anti-CD68 (1:200, ab303565, Abcam, Cambridge, MA), anti-CCL3 (1:200, ab277944, Abcam, Cambridge, MA), anti-CD11c (ab219799, Abcam, Cambridge, MA), anti-CD14 (1:1000, 17,000–1-AP, proteintec, Wuhan, China), anti-tenomodulin (1:200, ab203676, Abcam, Cambridge, MA). Biotin bound anti-mouse or anti-rabbit secondary antibodies and streptavidin horseradish peroxidase were used to detect primary antibodies. Antibody staining was visualized using the perkinlemer opal polychromatic IHC system according to the manufacturer’s protocol. The entire slide was scanned using the Vectra Polaris system (PerkinElmer), which initially captured the fluorescence spectra of five channels (DAPI, FITC, Cy3, Texas Red, and Cy5).

### Animal experiments

Animal experiments were approved by the Animal Ethics Committee of Fuwai Hospital. Animal experiments were designed according to the ARRIVE guidelines 2.0 [30] (Additional file 3). Cardiomyote-specific Dsg2 gene mutation (Dsg2^mut/mut^) mouse was produced by mating Myh6-cre mouse with Dsg-loxP mouse. Both Myh6-cre and Dsg-loxP mouse were purchased from Cyagen Bioscience. A total of 24 eight-week-old Dsg2^mut/mut^ male mice and age- and strain-matched wild type (WT) male mice were used in this study [31]. We performed genotypic and phenotypic assays to confirm the deletion of Dsg in the heart of mutant mice. Mice were maintained in a specific pathogen-free facility and provided free access to water and food. Mutant mice were continuously infused with the NLRP3 inflammasome inhibitor MCC950 [32] (5 mg/kg/day) or with PBS vehicle via subcutaneous alzet osmotic pump 2004 for four consecutive weeks. Randomization was used to allocate experimental units to control and treatment groups. Then, mice were anesthetized with isoflurane inhalation with 2 ~ 5% isoflurane for electrocardiogram and echocardiogram, and their hearts were harvested for histology and immunohistochemistry. Mouse feeding, injection of MCC950, and phenotype identification are handled by different individuals, and the person responsible for phenotype identification is not aware of the group information in advance. Two mice died in the experiment due to the arrhythmic events. The minimum number of mice in each group was not less than six.

Echocardiographic parameters were measured using a Vevo-2100 echocardiography system (VisualSonics Inc, Toronto, Canada) according to the American Society of Echocardiography guidelines [33]. Mice underwent two-dimensional transthoracic echocardiography at endpoint (after 4-week treatment). Appropriate concentration of isoflurane was used to maintain heart rate within 450–500 bpm. Parasternal short-axis views at the papillary muscle level were recorded, followed by the quantification of parameters of cardiac structure (left ventricular internal diameter and right ventricular area) and function (ejection fraction and fractional shorting). Measurements were obtained on at least five consecutive cardiac cycles. All the image analyses were conducted by an independent trained observer who was blinded to the experimental groups.

Electrocardiogram was recorded and analyzed according to published literatures [3, 34]. Anesthetized mice were fixed on a wooden board, followed by three electrodes inserted into the subcutaneous tissues of the left and right shoulders and the right hind leg. Then standard lead II electrocardiogram recordings (10 min for each mouse) were obtained with an electrocardiogram (ECG) processor (EP-2B, Softron Biotechnology, Beijing, China) and analyzed with a data acquisition program (SP2006, Softron Biotechnology, Beijing, China). Electrocardiographic parameters, including heart rate, P-wave amplitude, Q-wave amplitude, R-wave amplitude, and S-wave amplitude, were calculated using electrocardiograms lasting at least five consecutive heartbeats.

### Statistical analyses

R (version 3.6.1) was used for the statistical analysis. The Mann–Whitney *U* test by the Seurat (version 3.2.0) FindAllMarkers function was used to identify differentially expressed genes (DEGs) between the cell clusters. The cell ratios of each group were compared using logit transformation, followed by Student’s *t* test (two-sided with or without Welch’s correction) or Mann–Whitney *U* test between each group according to the results of normality test and variance homogeneity test. For differential expression and proportion, *p*-values were adjusted for multiple hypothesis testing using the Benjamini–Hochberg method. A *p*-value of < 0.05 was considered statistically significant.

## Results

### Cell atlas of blood and myocardium for ARVC patients

As NCMs were known to play pivotal roles in heart homeostasis and disorders [35, 36], we sought to determine their composition and the potential pathological cells in the heart with ARVC. We obtained 16 myocardium samples and 8 peripheral blood samples from 6 ARVC and 2 NC patients (left ventricle of ARVC [AC_LV], *n* = 6; right ventricle of ARVC [AC_RV], *n* = 6; PBMC of ARVC [AC_PBMC], *n* = 6; left ventricle of NC [NC_LV], *n* = 2; right ventricle of NC [NC_RV], *n* = 2; PBMC of NC [NC_PBMC], *n* = 2).

**Fig. 1:**
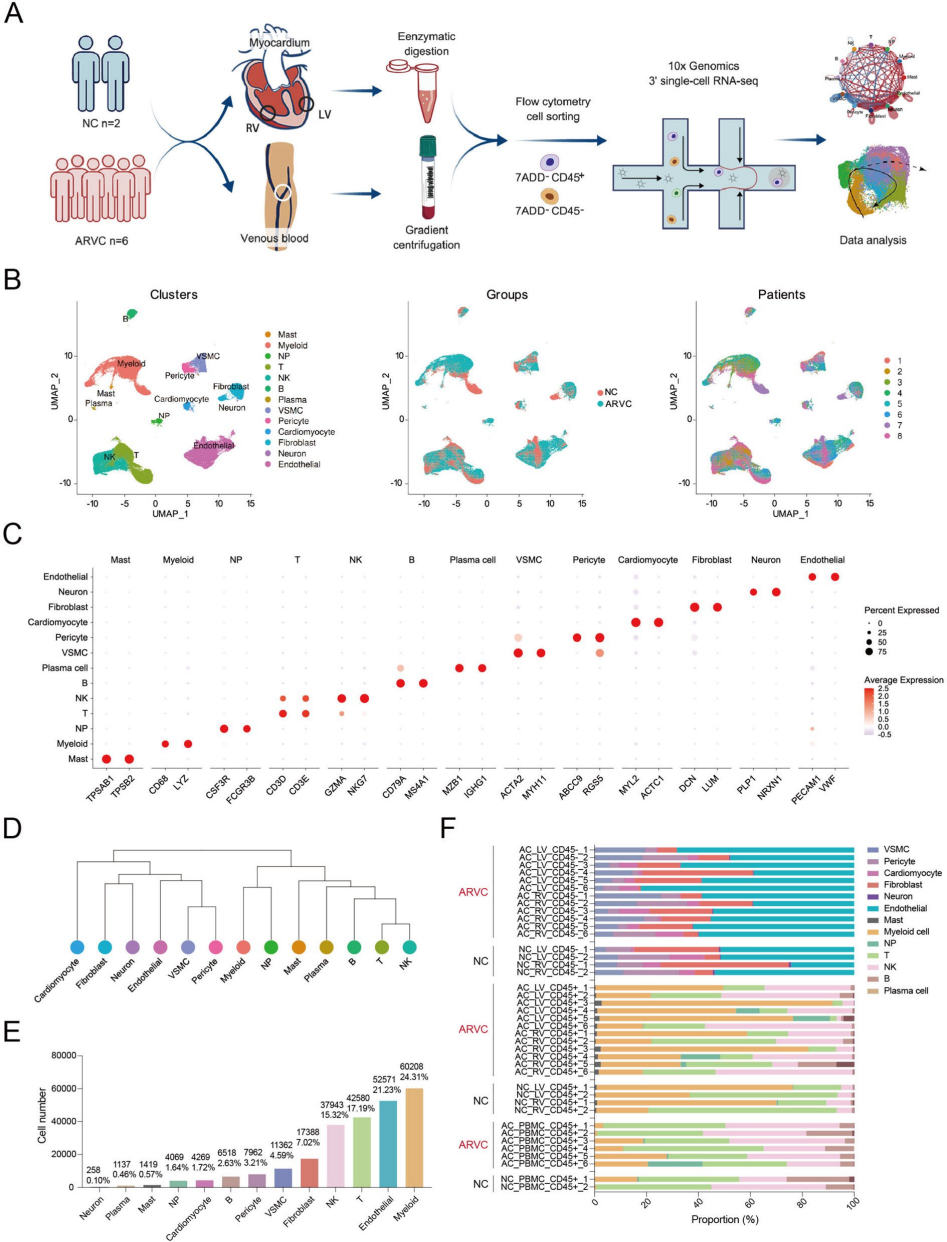
Overview of the 247,684 single cells isolated from ARVC and NC human hearts. **A** Workflow of the present study. **B** UMAP plots of the 247,684 cells colored according to the 13 major cell types, two phases, and 8 patients (left to right). **C** Expression of classic marker genes used to define the major cell types. **D** Dendrogram demonstrating the similarity of cluster centroids. **E** Cell numbers of each of the 13 major cell types. **F** Stacked bar plot depicting the cell-type composition of each sample. UMAP, uniform manifold approximation and projection; ARVC, arrhythmogenic right ventricular cardiomyopathy; NC, normal control; AC_LV, left ventricle of ARVC; AC_RV, right ventricle of ARVC; NC_LV, left ventricle of NC; NC_RV, right ventricle of NC; AC_PBMC, PBMC of ARVC; PBMC, peripheral blood mononuclear cell; NK, natural killer; NP, neutrophils; VSMC, vascular smooth muscle cell

The freshly collected myocardium were digested enzymatically to prepare the single-cell suspension, and the PBMCs were isolated by gradient centrifugation, and flow cytometry and cell sorting was performed to collect CD45^−^ and CD45^+^ cells from myocardium and CD45^+^ cells from PBMC. Then, the scRNA-seq was performed using the 10 × Genomics Chromium platform. The sequencing reads were demultiplexed, mapped to the GRCh38 human genome, and counted by unique molecular identifier (Fig. 1A).

After quality control (Additional file 2: Fig. S1, S2), we clustered a total of 247,684 cells (198,434 cells from ventricles and 49,250 cells from blood; 48,757 cells in NC and 198,927 cells in ARVC) and visualized the results using unsupervised clustering and uniform manifold approximation and projection (UMAP), respectively. Depending on the marker genes, a total of 13 cell types were identified (Fig. 1B, C; Additional file 2: Fig. S3). The marker genes for each cluster are available in a supplementary table (Additional file 4). Similar cell types were aggregated together based on transcriptional similarity, for example, vascular smooth muscle cell (VSMC) was aggregated pericyte, T cell was aggregated with natural killer (NK) cell. It was interesting that fibroblast was aggregated with neuron, providing clues for the potential origin of neurons, a minor population in human myocardium (Fig. 1D). Consistent with our previous work [12], the most abundant cell types were myeloid cells, endothelial cells, T cells, NK cells, and fibroblasts (Fig. 1E, F).

### CCL3^+^ myeloid cells accumulated in the right ventricle of ARVC patient

**Fig. 2:**
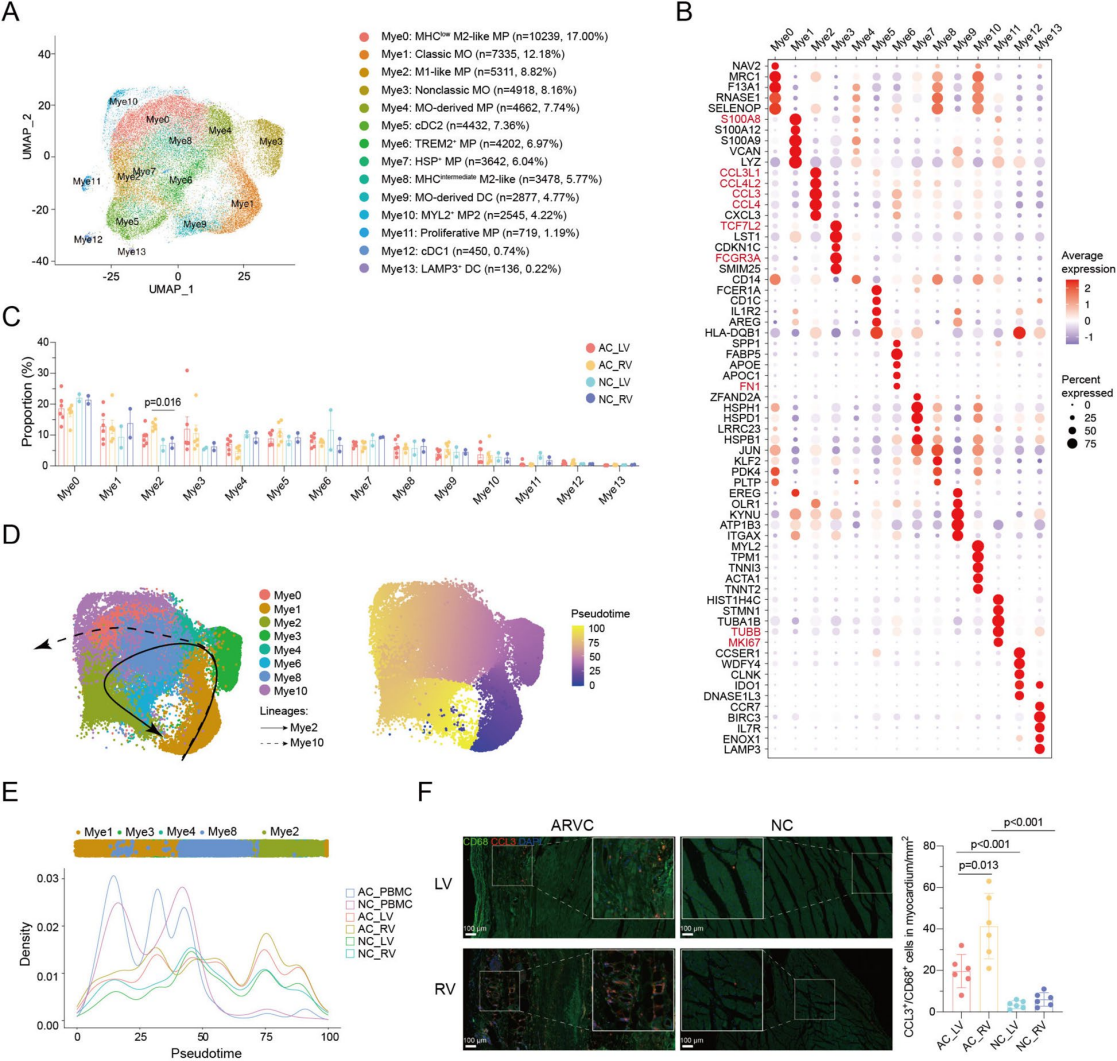
Myeloid subpopulations in ARVC human hearts.** A** UMAP embedding of 18 myeloid subpopulations. **B** Dot plot showing the top five marker genes of each subcluster. Dot color and size correspond to the expression of each gene and the proportion of cells expressing each gene, respectively. **C** The ratio of each subcluster in the different phases and topographic regions. **D**, **E** Trajectory analysis of selected clusters. **F** Multiple labeling staining for CCL3^+^ CD68^+^ macrophages; scale bar indicates 100 μm. Each spot represents one sample. Data are mean ± SD. Mann–Whitney *U* test was performed to compare the cellular ratio of Mye2 between ARVC and NC. UMAP, uniform manifold approximation and projection; ARVC, arrhythmogenic right ventricular cardiomyopathy; NC, normal control; AC_LV, left ventricle of ARVC; AC_RV, right ventricle of ARVC; NC_LV, left ventricle of NC; NC_RV, right ventricle of NC; AC_PBMC, PBMC of ARVC; PBMC, peripheral blood mononuclear cell; MP, macrophage; MO, monocyte; DC, dendritic cell; DEGs, differentially expressed genes. Gene names mentioned in the main text are color-coded

Myeloid cells were further classified into 14 subclusters (Mye0-13) comprising macrophages (MPs; including Mye0, 2, 4, 6, 7, 8, 10, and 11), monocytes (MOs, including Mye1 and 3), and dendritic cells (DCs; including Mye5, 9, 12, and 13) (Fig. 2A, B; Additional file 2: Fig. S4A-C). The marker genes for each cluster are available in a supplementary table (Additional file 5). Comparing the composition of myeloid subpopulations across patients, we observed a credible increase in the abundance of Mye2 in ARVC (AC_RV vs NC_RV, 2.14 fold, *P* = 0.016) (Fig. 2C; Additional file 2: Fig. S4C, D), which was consistent with previous reports [37]. Mye2 was identified as M1-like macrophage as it typically expressed proinflammatory genes like *IL1B*, *TNF*, and *CCL*. Mye11 was annotated as proliferated macrophage according to the top marker (*TUBB* and *MKI67*) and the function of nuclear division (Fig. 2B; Additional file 2: Fig. S4B, 4C). The accumulation of proinflammatory macrophage (Mye2) in myocardium with ARVC was assistant with previous report that the presence of inflammatory cell infiltration have been found in up to two-thirds of ARVC hearts [38]. We further found that Mye2 was more abundant in AC_RV when compared with AC_LV (Fig. 2C). The proinflammatory genes such as *IL1B*, *IL6*, and *TNF* were significantly upregulated in AC_RV when comparing to AC_LV and NC_RV (Additional file 2: Fig. S5A). Moreover, *IL1B* and *TNF* were mainly expressed in myeloid cells, predominantly in Mye2 (Additional file 2: Fig. S5B, S6B).

To reveal the possible pseudotime trajectory of Mye2, we constructed a trajectory analysis by using slingshot^15^ and observed two distinct trajectories: Mye1 connected with Mye3, Mye4, and Mye8, which subsequently branched into two different lineages, i.e., Mye10 and Mye2 (Fig. 2D), which was consisted with monocle2 results (Additional file 2: Fig. S5C). Along the Mye2 lineage, the AC_RV-cells were enriched toward the end while AC_PBMC-cells were enriched in the origin (Fig. 2E). Classical monocyte markers such as *S100A8* and *FCN1* were downregulated, non-classical monocyte markers such as *FCGR3A* and *TCF7L2* were gradually upregulated and then downregulated, and Mye2 markers such as *CCL3* and *CCL4* were upregulated (Additional file 2: Fig. S5D), confirming that Mye2 originated from classical monocyte and enriched in AC_RV along the Mye2 lineage. Immunostaining results showed a 2.21-fold and 5.13-fold increase in *CCL3*^+^ macrophages (Mye2) cell ratio in samples from AC_RV myocardium compared to AC_LV (*P* = 0.013) and NC_RV (*P* < 0.001), respectively (Fig. 2F).

### Activated fibroblasts in myocardium of ARVC patient

**Fig. 3:**
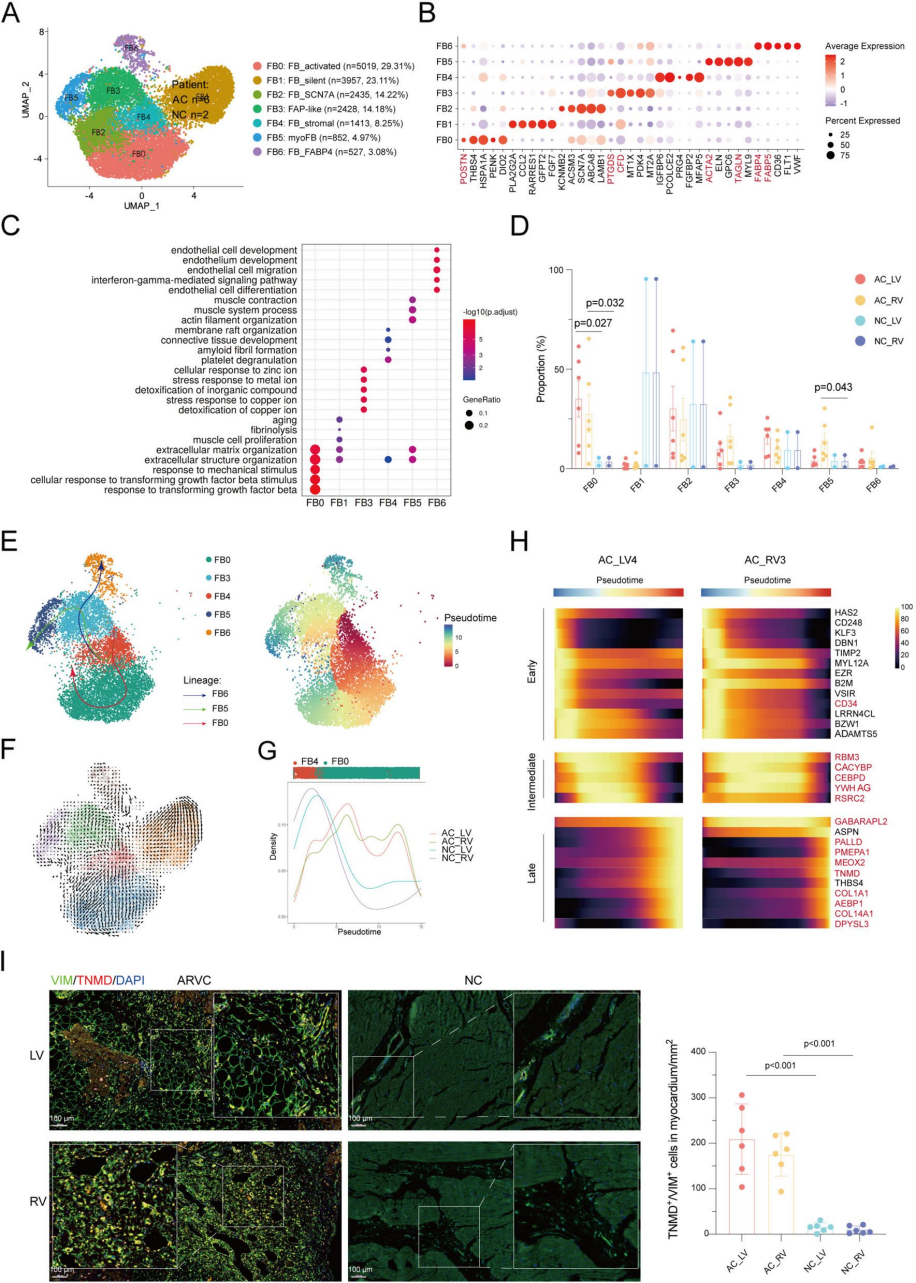
Fibroblast subpopulations in ARVC human hearts.** A** UMAP embedding of eight fibroblast subpopulations. **B** Dot plot showing the top five marker genes of each subcluster. Dot color and size correspond to the expression of each gene and the proportion of cells expressing each gene, respectively. **C** The top five enriched GOBP of each cluster. **D** The ratio of each subcluster in the different phases and topographic regions. **E** Trajectory analysis of selected fibroblast clusters based on Slingshot. **F** RNA velocity of fibroblast clusters. **G** Density plots reflecting the number of FB cells along the lineage FB0 stratified for different phases and topographic regions. **H** Predicted expression of genes showing interesting patterns based on a negative binomial generalized additive model (NB-GAM) for each gene across pseudotime in patient AC_LV_4 (left) and patient AC_RV_3 (right). Normalized expression is smoothed expression from NB-GAM scaled to the maximum value for each gene. **I** Multiple labeling staining for TNMD^+^ VIM^+^ fibroblasts; scale bar indicates 100 μm. Each spot represents one sample. Data are mean ± SD. Mann–Whitney *U* test was performed to compare the cellular ratio of TNMD^+^ VIM^+^ fibroblasts between ARVC and NC. FAP, fibro-adipogenic progenitor; FB, fibroblast; ARVC, arrhythmogenic right ventricular cardiomyopathy; NC, normal control; AC_LV, left ventricle of ARVC; AC_RV, right ventricle of ARVC; NC_LV, left ventricle of NC; NC_RV, right ventricle of NC; AC_PBMC, PBMC of ARVC. Gene names mentioned in the main text were color-coded

Fibroblasts were further split into seven subclusters (FB0-6, Fig. 3A). FB0 was identified as activated fibroblast as it typically expressed pro-fibrosis gene *POSTN* and the function of response to TGFβ (Fig. 3B, C). The highest expression of pre-adipocyte marker *CFD* [13] and fibro-adipogenic progenitor (FAP) marker *PDGFRA* indicating that FB3 was FAP-like. FB4 was annotated as stromal fibroblast as its higher expression of *CD34*. FB5 was annotated as myofibroblast according to the contractile marker (*ACTA2* and *TAGLN*). FB6 highly expressed adipocyte markers such as *FABP4* and *FABP5* (Fig. 3B; Additional file 2: Fig. S6A). The marker genes for each cluster are available in a supplementary table (Additional file 6).

Comparing the composition of fibroblast subpopulations between ARVC and NC, we observed a statistically credible increase in the abundance of FB0 (AC_RV vs NC_RV, 4.29 fold, *P* = 0.032; AC_LV vs NC_LV, 5.21 fold, *P* = 0.027) and FB5 (AC_RV vs NC_RV, 2.46 fold, *P* = 0.043) in ARVC (Fig. 3D; Additional file 2: Fig. S6B). Slingshot revealed additional phenotypic heterogeneity and identified three trajectories, namely FB0, FB5, and FB6 lineages (Fig. 3E). Briefly, FB4 connected closely with FB3, which branched-off into two different trajectories to form FB5 lineage and FB6 lineage. FB4 originally formed FB0 lineage as well. RNA velocity analysis confirmed these trajectories (Fig. 3F). Consistent with the greater abundance of FB0 in the AC_LV and AC_RV compared with the NC_LV and NC_RV, FBs in the AC_LV and AC_RV were enriched toward the end of FB0 (Fig. 3G). The starkest change in cell-type composition observed in ARVC was FB0 that was found almost exclusively in patient with ARVC, especially in AC_LV_4 and AC_RV_3 (Additional file 2: Fig. S6C). To better understand the gradient of expression between quiescent and activated fibroblasts, we extracted cells of patient AC_LV_4 and AC_RV_3 from FB0 lineage and performed a trajectory analysis. Several genes, including pro-fibrosis genes *AEBP1*, *COL1A1*, and *COL14A1* and genes rarely studied in the field of fibrosis (*GABARAPL2*, *PALLD*, *PMEPA1*, *MEOX2*, *TNMD*, and *DPYSL3*) showed increased expression across the trajectory, whereas *CD34* showed decreased expression across the trajectory. Notably, a subset of genes including *RBM3*, *CACYBP*, *CEBPD*, *YWHAG*, and *RSRC2* showed increased expression at various stages along the gradient (Fig. 3H). Immunostaining results showed a 14.25-fold increase in *TNMD*^+^ fibroblast cell ratio in samples from AC_RV myocardium compared to NC_RV (*P* < 0.001) and a 10.83-fold increase in *TNMD*^+^ fibroblast cell ratio in samples from AC_LV myocardium compared to NC_LV (*P* < 0.001) (Fig. 3I).

Interestingly, FBs in the ARVC were especially enriched toward the end of FB5 and FB6 (Additional file 2: Fig. S6D). Along the FB5 lineage, FAP-like genes (*CFD*, *PDGFRA*, and *PTGDS*) increased halfway through the trajectory, but then decreased. Contractile genes (*ACTA2*, *TAGLN*, and *MYL9*) were upregulated along the lineage (Additional file 2: Fig. S6E). Immunofluorescence result showed a 4.13-fold increase in FB5 cell ratio from AC_RV compared with NC_RV (*P* < 0.001) and a 2.77-fold increase in FB5 cell ratio from AC_RV compared with NC_RV (*P* < 0.001) (Additional file 2: Fig. S6F).

### Fibroblasts may partially contribute to the cardiac arrhythmia of ARVC

**Fig. 4:**
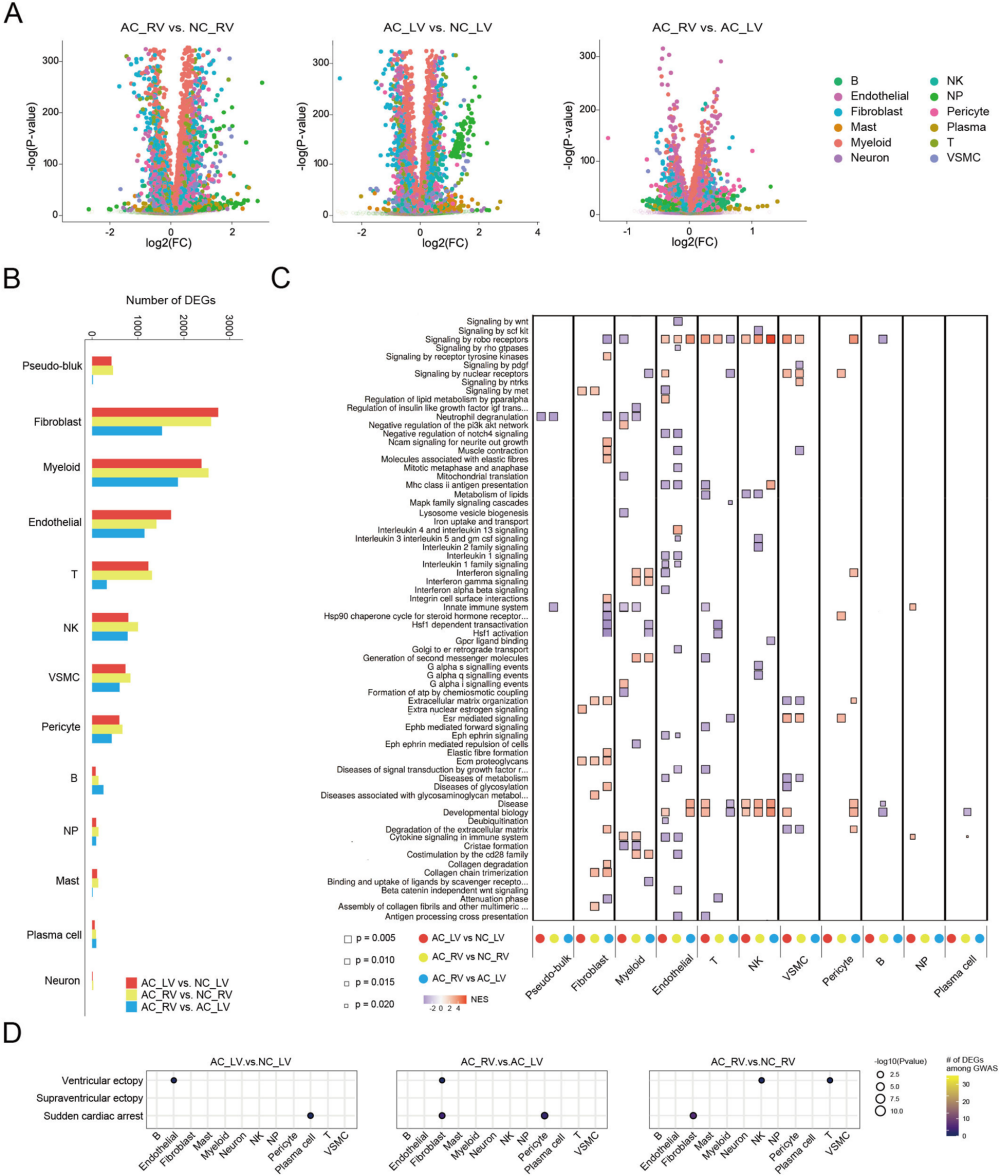
Fibroblasts may partially contribute to the cardiac arrhythmia of ARVC.** A** Log-fold-change and two-sided *P*-value for expression changes between AC_RV (*n* = 6) and NC_RV (*n* = 2) (left), AC_LV (*n* = 6) and NC_LV (*n* = 2) (center), and AC_RV and AC_LV (right) hearts for each gene tested using limma–voom differential expression analysis. Genes are colored by cell type with larger, opaque dots representing genes with FDR < 0.01 based on the Benjamini–Hochberg procedure. **B** The number of significantly differentially expressed genes (FDR < 0.01) by cell type for each comparison in (**A**). **C** Reactome pathway enrichment for differential expression between each comparison in (**A**) by cell type. The size of each square represents a two-sided *P*-value from GSEA and shading represents the normalized enrichment score (NES). Only pathways with a Benjamini–Hochberg FDR < 0.05 in both the GSEA and hypergeometric test for over-representation in at least one cell type are shown. Pathways with FDR < 0.05 in the GSEA test are denoted with a black outline. **D** Dot plot showing the number of DEGs per cell type that overlaps as GWAS risk variants across cardiac arrhythmia traits from the GWAS catalog (NHGRI-EBI) [39]. Significance of overlap is based on FDR < 0.05. GSEA, gene set enrichment analysis; NES, normalized enrichment score; FDR, false discovery rate; ARVC, arrhythmogenic right ventricular cardiomyopathy; NC, normal control; AC_LV, left ventricle of ARVC; AC_RV, right ventricle of ARVC; NC_LV, left ventricle of NC; NC_RV, right ventricle of NC; AC_PBMC, PBMC of ARVC; PBMC, peripheral blood mononuclear cell; NK, natural killer; NP, neutrophils; VSMC, vascular smooth muscle cell; DEGs, differentially expressed genes

To identify key changes in ARVC tissues, we compared data between AC_RV and NC_RV, AC_LV and NC_LV, and AC_RV and AC_LV tissues at the level of pseudo-bulk and each cell type. Substantial differences were observed in transcriptional level (Fig. 4A). Notably, the largest number of DEGs between each of the ARVC and NC group (false discovery rate (FDR) < 0.01) was found in fibroblasts, suggesting that the largest transcriptional differences occur in this cell type. The marker genes for each cluster are available in Additional file 7. There was also a huge set of genes that were differentially expressed between AC_RV and AC_LV hearts (Fig. 4B). Next, we performed pathway enrichment analyses by cell type to identify any systematic patterns in gene dysregulation (Fig. 4C). In general, similar pathway enrichments were observed in AC_RV versus NC_RV and AC_LV versus NC_LV DEGs. Of note, neuron showed no systematic up- or downregulation in patients with AC. Conversely, we observed robust dysregulation in ARVC of several pathways in myeloid, including cytokine signaling in immune system, neutrophil degranulation, and signaling by robo receptors. Similarly, multiple pathways in endothelial exhibited dysregulation among ARVC hearts, including cytokine signaling in immune system, interleukin 1 signaling, and MHC II antigen presentation. There were specific pathways dysregulated in AC_RV, including co-stimulation of the CD28 family, interferon signaling and generation of secondary messenger molecules in myeloid, collagen chain trimerization, and ECM organization in fibroblasts.

To further determine the enrichment of ARVC DEGs within genetic variants associated with complex traits and diseases in a cell-type-specific fashion, we obtained genome-wide association study (GWAS) summary statistics for cardiac arrhythmia traits (Additional file 1: Table S5). We found a strong enrichment of fibroblast DEGs residing within GWAS hits of cardiac arrhythmia traits, especially in sudden cardiac arrest (Fig. 4D). Together, these data suggest that fibroblasts may partially contribute to the cardiac arrhythmia of ARVC.

### Predicted and altered cell–cell interactions in the heart of ARVC patient

**Fig. 5:**
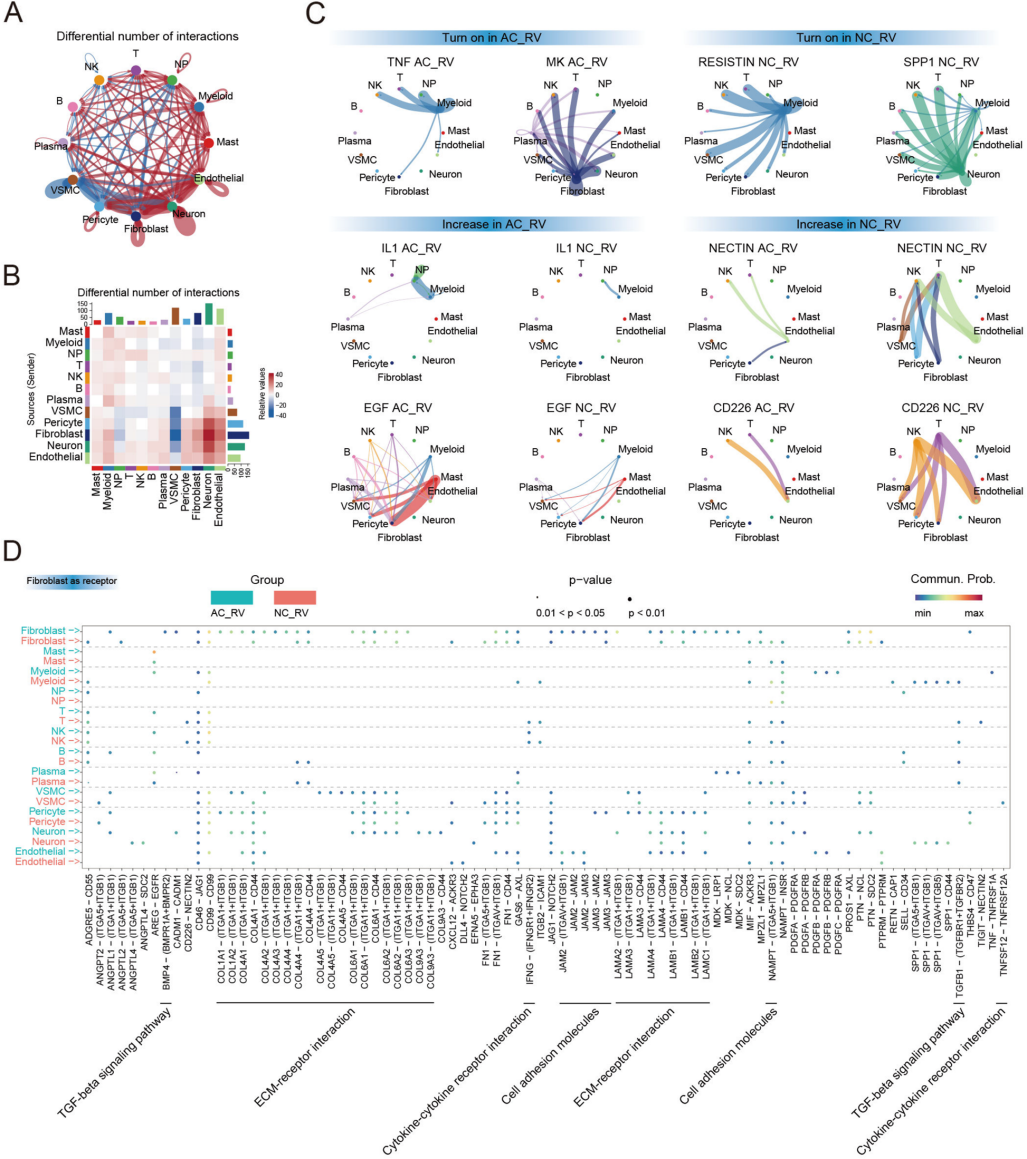
Predicted and altered cell–cell interactions in ARVC patient hearts.** A** Net plot showing the interaction number and weight among the 12 major cell clusters. Each dot indicates one cell cluster and its size is proportional to the number of cells in the cluster. The thickness of the lines connecting cell clusters indicates the differential interaction number (blue line indicates an increase in NC_RV, red line indicates an increase in AC_RV). **B** Differential number of the cellular interactions between NC_RV and the AC_RV. Red and blue represent enrichment in the AC_RV and NC_RV, respectively. **C** Circos plots showing the inferred intercellular communication network among the major cell types. **D** Bubble plot showing the selected ligand–receptor interactions between the 12 major cell types (ligand) and fibroblasts. ARVC, arrhythmogenic right ventricular cardiomyopathy; NC, normal control; AC_LV, left ventricle of ARVC; AC_RV, right ventricle of ARVC; NC_LV, left ventricle of NC; NC_RV, right ventricle of NC; AC_PBMC, PBMC of ARVC; PBMC, peripheral blood mononuclear cell; NK, natural killer; NP, neutrophils; VSMC, vascular smooth muscle cell

By examining the expression of genes encoding for receptors and ligands, we inferred intercellular signaling and communication [20]. We initially quantified the probability of cell–cell interactions and compared signaling between cell types, and then aggregated information to produce cell-specific and across all-cell-type data for ARVC relative to controls. This sequential approach accounted for differential abundances of cell states. Compared with NC_RV, the number of interactions in AC_RV were increased (Fig. 5A), especially the fibroblast-neuron interaction (Fig. 5B). We detected aberrant intercellular signaling across disease (Fig. 5C), including upregulation of the proinflammatory TNF, MK [40], and IL1 pathway. Signaling depending on EGF was also increased in disease, implying pro-fibrosis BMP, FN1, collagen, EGF, IGF, and TGF pathways that promote fibrosis. Signaling dependent on VEGF, NOTCH, and ANGPT was also increased in disease, implying vascular remodeling. Aberrant intercellular signaling pathways were also identified between NC_LV and AC_LV, as well as AC_LV and AC_RV (Additional file 2: Fig. S7): Comparing to NC_LV, signaling such as pro-fibrosis TGFβ, and pro-inflammation MK, TNF, and IL1 were upregulated in AC_LV. Comparing to AC_LV, signaling such as HSPG, CCL, BAFF, and CD86 were upregulated in AC_RV.

Since fibroblasts may partially contribute to the cardiac arrhythmia of ARVC (Fig. 4D), we focused on the influences from each cell type to fibroblast. To identify the differences between NC_RV and AC_RV, we filtered ligands-receptors which were only enriched in NC_RV or AC_RV (Fig. 5D). Fibroblasts in AC_RV especially received TNF/TNFSF12 from myeloid cells. Besides, the ECM-receptor interactions of fibroblasts with VSMC, neuron, endothelial cells, and fibroblasts were strongly enriched.

### Pharmacological inhibition of proinflammatory macrophages significantly alleviate the right ventricular dysfunction in a ARVC mouse model

Due to the enrichment and possible cell–cell interaction of Mye2 in AC_RV (Fig. 2; Additional file 2: Fig. S8), we explored whether inhibition of Mye2 could rescue the phenotype of ARVC mouse model. *NLRP3* was identified as the potential target because results showed it was one marker of Mye2 (Additional file 5), and studies suggested it played a key role in inflammatory response through the processing and release of IL-1β and the induction of cell death processes [41]. *NLRP3*, coding a cytosolic multiprotein complex, was preferentially expressed in myeloid cells, especially in subcluster Mye2 and other three subclusters (Mye1, Mye5, and Mye9) (Additional file 2: Fig. S9A, B). Similar to the enrichment of Mye2 in AC_RV (Fig. 2G), *NLRP3* was expressed at the highest levels on AC_RV when analyzing all myeloid cells or subclusters (Mye2, Mye1, Mye5, and Mye9) (Additional file 2: Fig. S9C-G). What is more, we observed the consistent trend of *NLRP3* and proinflammatory genes *IL1B* and *CXCL8* along the Mye2 lineage (Additional file 2: Fig. S9H). Further, the expression level of *NLRP3* positively correlated with *IL1B* and *CXCL8* among myeloid subclusters (Pearson *r* = 0.812, *P* < 0.001, and Pearson *r* = 0.856, *P* < 0.001, respectively) (Additional file 2: Fig. S9I, J). These results suggested NLRP3 could be one potential target to treat ARVC by alleviating the inflammatory effects of myeloid cells, especially for proinflammatory Mye2.

**Fig. 6:**
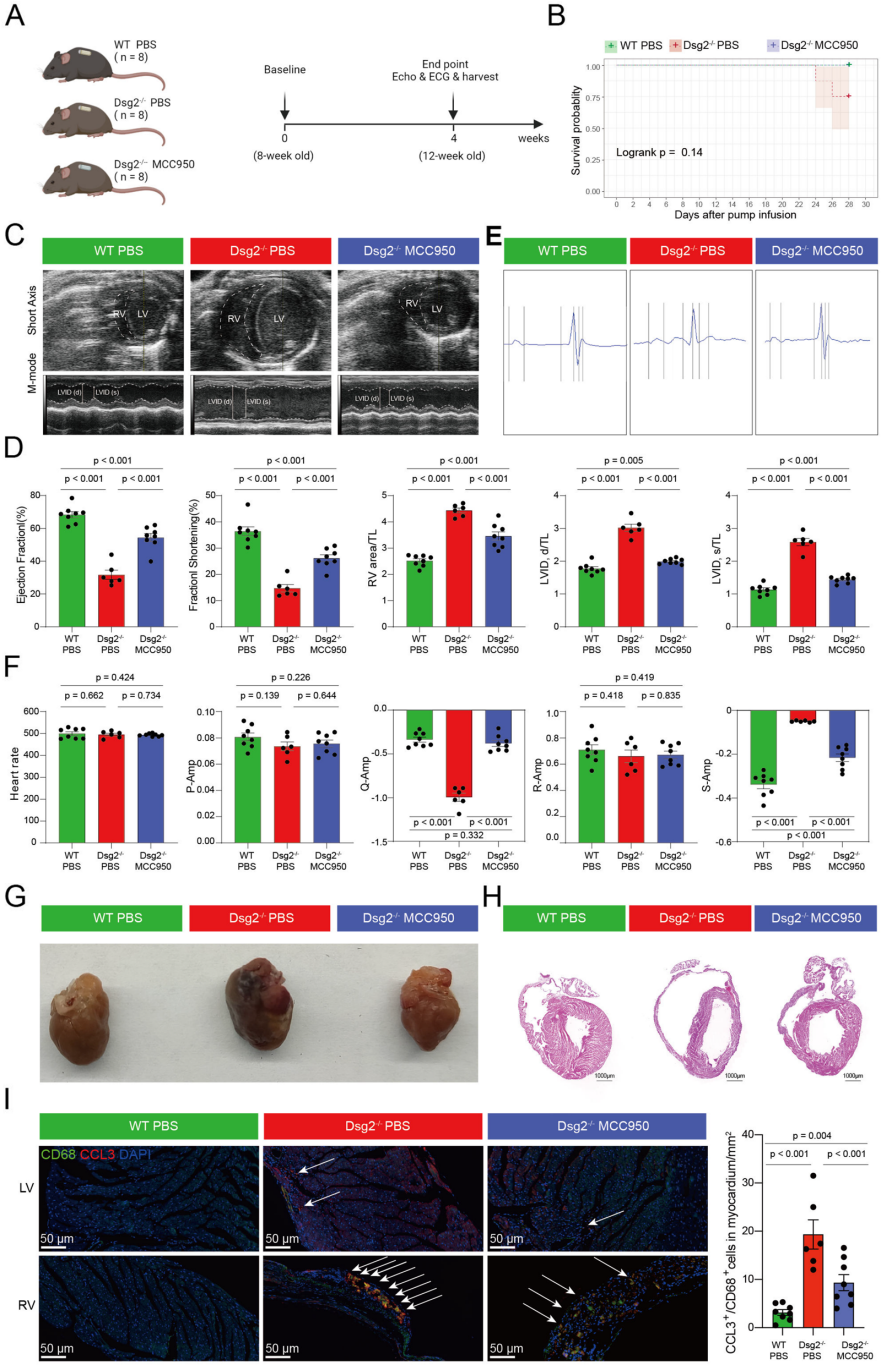
Pharmacological inhibition of proinflammatory macrophages significantly alleviate the right ventricular dysfunction in a ARVC mouse model.** A** Experimental protocol. **B** Survival curve.** C** Representative short-axis M-mode Echo from PBS-infused wild type and Dsg2^mut/mut^ mice, and MCC950-infused Dsg2^mut/mut^ mice at 12 weeks of age. **D** Echo of PBS-infused wild type and Dsg2^mut/mut^ mice, and MCC950-infused Dsg2^mut/mut^ mice at 12 weeks of age. **E**, **F** Representative signal-averaged electrocardiograms (**E**) and electrocardiogram parameters (**F**) of PBS-infused wild type and Dsg2^mut/mut^ mice, and MCC950-infused Dsg2^mut/mut^ mice at 12 weeks of age. **G**, **H** Representative gross pathology (**G**) and long-axis sections of the hearts stained with H&E-stained (**H**) micrographs from PBS-infused wild type and Dsg2^mut/mut^ mice, and MCC950-infused Dsg2^mut/mut^ mice at 12 weeks of age. Scale bar, 1000 µm. **I** Multiple labeling staining for CCL3^+^ CD68^+^ macrophages; scale bar indicates 100 μm. Each spot represents one sample. Data are mean ± SD. The Mann–Whitney *U* test was performed to compare the cellular ratio of CCL3^+^ CD68^+^ macrophages.TL, tibial length; LVIDs, left ventricular internal diameter at end-diastole; LVIDs, left ventricular internal diameter at end-systole; P-Amp, P-wave amplitude; R-Amp, R-wave amplitude; Q-Amp, Q-wave amplitude; S-Amp, S-wave amplitude

Then we explored the effects of NLRP3-targeted therapy in a ARVC model by infusing MCC950, a diarylsulfonylurea-containing compound that was shown to selectively inhibit the oligomerization and activation of the NLRP3 inflammasome in response to canonical and non-canonical stimuli [32]. Dsg2^mut/mut^ mice were treated with MCC950 by continuous infusion over a 4-week period beginning when the mice were 8 weeks of age [42] (Fig. 6A). Approximately 25% (2 of 8) of the PBS-infused Dsg2^mut/mut^ mice died because of cardiac failure or arrhythmias, whereas none of the PBS-infused WT mice or MCC950-treated Dsg2^mut/mut^ mice died (Fig. 6B). Compared to PBS-infused WT mice, PBS-infused Dsg2^mut/mut^ mice display apparently impaired left ventricular function (ejection fraction, 31.52% vs 68.31%, *P* < 0.001; fraction shortening, 15.16% vs 36.82%, *P* < 0.001; diastolic LVID/tibial length, 3.02 vs 1.74, *P* < 0.001; systolic LVID/tibial length, 2.64 vs 1.13, *P* < 0.001), right ventricular dilatation (RV area/tibial length, 4.52 vs 2.47, *P* < 0.001), electrocardiographic abnormalities like Q- and S-wave amplitude (Q-wave, − 0.97 vs − 0.24, *P* < 0.001; S-wave, − 0.06 vs − 0.35, *P* < 0.001), increased cardiac fibrosis (3.18% vs 0.57%, *P* < 0.001), and a marked accumulation of proinflammatory macrophages, CD14^+^ monocytes, and CD11c^+^ DC cells in myocardium (19.16 vs 3.51/mm^2^, *P* < 0.001; 23.15 vs 3.24/mm^2^, *P* < 0.001; 5.33 vs 2.16/mm^2^, *P* < 0.001, respectively). In contrast, those changes were alleviated in MCC950-treated Dsg2^mut/mut^ mice (Fig. 6C–I; Additional file 2: Fig. S10). Overall, compared with PBS-treated Dsg2^mut/mut^ mice, MCC950-treated Dsg2^mut/mut^ mice exhibited better biventricular function (ejection fraction, 56.33% vs 31.52%, *P* < 0.001; fraction shortening, 27.29% vs 15.16%, *P* < 0.001; diastolic LVID/tibial length, 1.97 vs 3.02, *P* < 0.001; systolic LVID/tibial length, 1.52 vs 2.64, *P* < 0.001), less right ventricular dilatation (RV area/TL, 3.38 vs 4.52, *P* < 0.001), decreased cardiac fibrosis (2.04% vs 3.18%, *P* < 0.001), and less proinflammatory macrophages, CD14^+^ monocytes, and CD11c^+^ DC cell infiltration compared with PBS-infused Dsg2^mut/mut^ mice (3.51 vs 9.27/mm^2^, *P* < 0.001; 8.22 vs 23.15/mm^2^, *P* < 0.001; 3.34 vs 5.33/mm^2^, *P* < 0.001).

Together, these results suggest that pharmacological inhibition of *NLRP3* may inactivate myeloid cell subpopulation associated with ARVC (Mye2) to solve to the inflammation and fibrosis of ARVC.

## Discussion

**Fig. 7:**
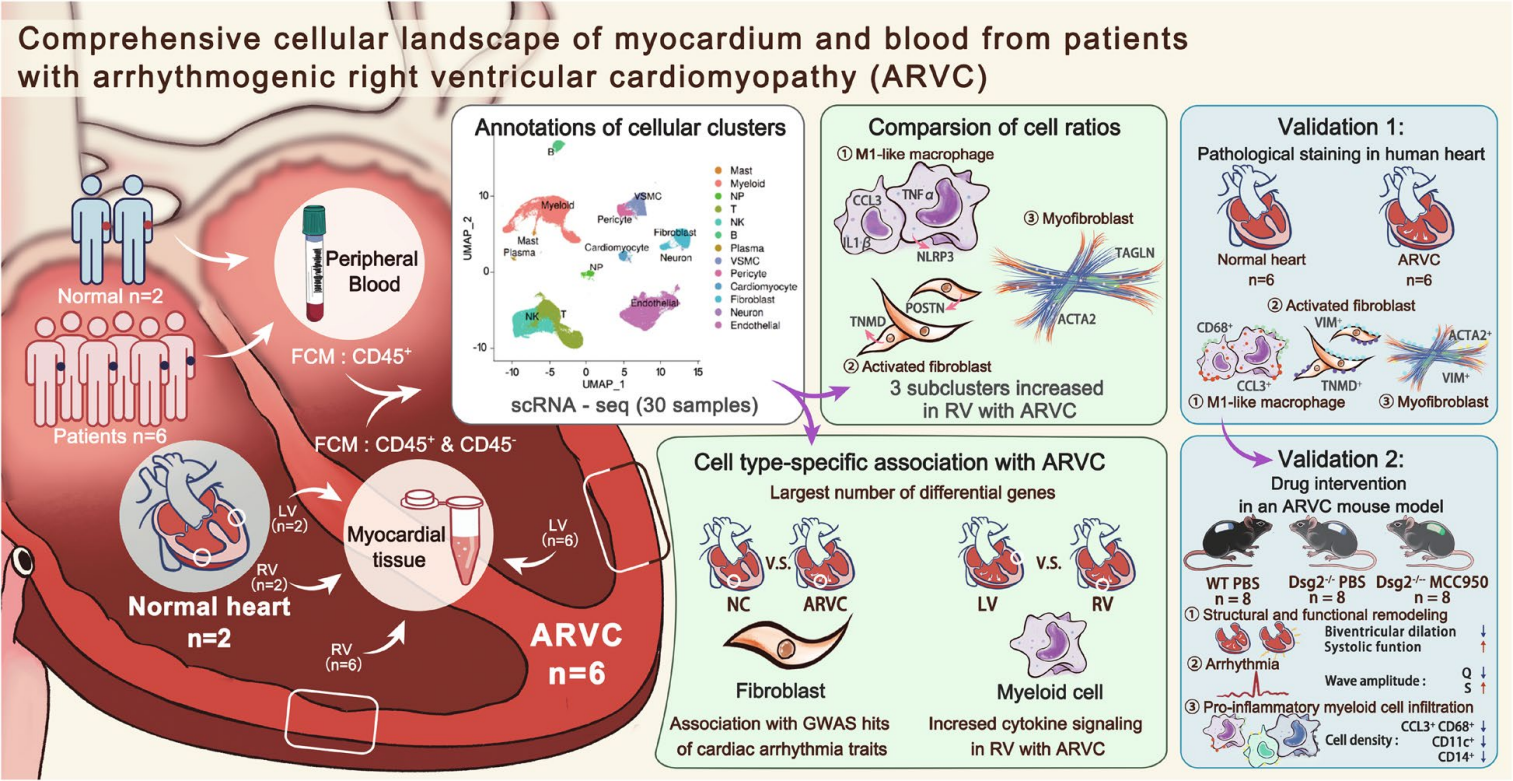
Summary of the present study. We constructed a comprehensive cellular landscape of human biventricular myocardium and blood from 6 end-stage heart patients with ARVC and 2 normal controls by using single-cell RNA sequencing. We identified the increased cell ratios of M1-macrophage, activated fibroblast, and myofibroblast in right ventricle with ARVC compared to normal control. The cell type-specific association with ARVC were also described. To sure robust science, we used two validation methods of the key findings of single-cell RNA sequencing data, including pathological staining in human heart with a larger sample size and drug intervention in an ARVC mouse model. ARVC, arrhythmogenic right ventricular cardiomyopathy

Understanding cellular-specific regulatory changes under diseased conditions is of fundamental importance for successful drug development in case lacking effective drug treatment for ARVC. In this study, cells from blood and biventricular myocardium of ARVC were sequenced by scRNA-seq to define specific changes in expression profile, subpopulation composition, and intercellular communication (Fig. 7). By comparing gene expression profiles from ARVC patients’ ventricles, we were able to show an enrichment for known and markers of cell changes induced in ARVC condition. Based on this heterogeneity, we bioinformatically clustered transcriptionally related cells or genes, each of which is likely related to different cellular functions (Additional file 1: Table S6). In addition, based on scatter properties of ARVC cells, we were able to determine the enrichment of ARVC DEGs within genetic variants associated with complex traits and diseases in a cell-type-specific fashion. Previous single-nucleus/cell studies on cardiac tissues may report different numbers of cardiomyocyte or noncardiomyocyte subpopulations of ARVC due to different resolutions applied for clustering across studies including human and animal model [16, 43, 44]. However, our report enrolled different tissues of ARVC patients including blood samples and both ventricles which was the most complete sample in one study as known, and all the reports and ours suggested that the transcriptomic states of cells in human hearts and blood were continuous rather than discrete.

While the mechanism of ARVC is orchestrated by multiple cell types, among which immune cells have attracted more and more attention that inflammatory signaling is activated in ARVC and drives key features of the disease [3, 6, 45, 46]. As such, pharmacological interventions that directly target immune cells may be the most promising strategy for alleviating pathological arrhythmia or mitigating the progression in ARVC [3]. Proinflammatory myeloid subpopulation in ARVC patient hearts was found to play essential roles in the pathogenesis of ARVC as previous report that the presence of inflammatory infiltrates in more than two-thirds of ARVC hearts [38]. According to the cellular function, we found that myeloid cells included classified 14 subclusters of myeloid cells comprising MPs, MOs, and DCs. As for MPs, 8 subpopulations were included: *CCL3*^+^ MPs, *MHCII*^low^ MPs, MO-derived MPs, *TREM2*^+^ MPs, *MHCII*^int^ MPs, *MYL2*^+^ MPs, proliferation MPs, and *HSP*^+^ MPs. Some of these MP clusters have been reported in our previous study about human heart failure, such as *TREM2*^+^ MPs, proliferation MPs, and MHCII-associated MPs (*MHCII*^low^ and *MHCII*^int^) [12], which indicated that these cell clusters participated in the process of heart failure. Heart failure was one of characteristics of ARVC. *CCL3*^+^ MPs (Mye 2) was not reported in the pathogenesis of ARVC before, and the cell density plot reflecting the AC_RV-cells were enriched toward the end of *CCL3*^+^ MPs from AC_PBMC-cells (Fig. 2D). Besides, *CCL3* and *CCL4* were upregulated in this clusters identified as proinflammatory myeloid subpopulation, consistent with that the two genes were markers of myeloid chemotaxis and activation [47]. The NLRP3 inflammasome is a macromolecular platform that senses tissue injury and, in response, processes active IL-1β (upregulation in *CCL3*^+^ MPs) and IL-18, two major proinflammatory cytokines, and could induce an inflammatory form of cell death termed pyroptosis, participating in inflammatory disease such as Alzheimer disease, inflammatory bowel disease, experimental autoimmune encephalitis, myocardial infarction, and so on [48]. Pharmacologically targeting NLRP3 with novel small molecules has become a potential strategy for treatment of inflammatory diseases. In our study, we found that MCC950, an inhibitor of NLRP3 which could attenuate the severity of experimental autoimmune encephalomyelitis, could significantly alleviate the cardiac inflammatory, cardiac function and arrythmia of Dsg2^mut/mut^ mice exhibiting better biventricular function, less left ventricular diameter, and less proinflammatory CCL3^+^ macrophage infiltration (Fig. 6). Our experimental evidence indicated that alleviating right ventricular dysfunction phenotype in mice by a reduction in CCL3^+^ CD68^+^ cells in heart tissue. This study and our previous work have demonstrated an associated between PBMC and cardiac myeloid cells [12], so the question that inhibiting the recruitment of proinflammatory macrophages into the heart also have an ameliorative effect need to be explored in the future. Although the recruitment mechanism of proinflammatory macrophages into heart was complex, some chemokines such as CCL5 was reported to play roles in disease pathogenesis and its antibody could inhibit the progression of some immune diseases like multiple sclerosis [49]. We also found that proinflammatory macrophages expressed high CCL3, which maybe a potential target to inhibit the recruitment of proinflammatory macrophages in heart. These observations might provide further evidence that ARVC disease alleles (DSG2) activated an innate immune response that was independent of the actions of professional inflammatory cells associated with heart failure. More, pharmacological inhibition of NLRP3 activation results in potent therapeutic effects in a wide variety of rodent models of inflammatory diseases [50, 51], which have been taken into consideration in clinic. More approaches should be performed such as genetic disruption and another NLRP3 inhibitor in the future. Besides, it was meaningful for ARVC drug treatment due to lack of efficient drug treatment, but more investigations were needed to support in the future.

Fibrosis was reported to be a common response to cardiomyocyte injury in the heart, and ARVC was no exception [52]. Unfortunately, the molecular mechanism of recruiting fibrofatty tissue to the damaged myocardium remained poorly known. A complex network of interactions promoting cardiac fibrosis was to be regarded by investigation into scar formation after myocardial infarction and diabetes-mellitus-induced cardiac fibrosis. It was reported that myocardial fibrosis predicts ventricular arrhythmias and sudden death, which were the leading cause of death in ARVC [53]. Based on the GWAS analysis, we found a strong enrichment of fibroblasts DEGs residing within GWAS hits of some cardiac arrhythmia traits, especially sudden cardiac arrest. Fibroblast activation was an inducer of cardiac fibrosis. Understanding the regulatory mechanism underlying fibroblast activation in ARVC was critical for developing effective medical therapies to alleviate cardiac fibrosis and, as a result, prevent adverse outcomes including sudden cardiac death in ARVC patients. The activated fibroblast cluster FB0 was found to be significantly expanded in ARVC (Fig. 3D). In order to reveal the mechanism of activated fibroblasts, a total of 11 potential key genes were identified to contribute into the activation of fibroblasts. Among them, *AEBP1* has been demonstrated to be associated with fibrosis in DCM [12] and *THBS4* has also been demonstrated to promote skin fibrosis [54]. In our study, *TNMD* was found to be upregulated in fibroblasts of ARVC. *TNMD* was the ECM-associated gene and increased in human visceral adipocytes [55]. The association between proinflammatory immune cells such as macrophages and fibroblast has been reported in many disease including cancer [56], cardiovascular disease [12, 57], and aging [58]. In our study, we found that fibroblasts in AC_RV especially received TNF/TNFSF12 from proinflammatory macrophages (Mye2), indicating that proinflammatory macrophages strongly interact with fibroblast with the largest number of ligand–receptor pair in AC_RV (Fig. 5B). Besides, we found that fibroblasts could also interact with macrophages through MK (Fig. 5C), MK was reported as proinflammatory signaling pathway [59]. Together, these results indicated that the relationship of macrophage and fibroblast was more complex than we expected. And inhibiting NLRP3 by MCC950 could ameliorate the phenotype of ARVC including fibrosis, which also support the relationship between proinflammatory and fibroblasts (Additional file 2: Fig. S10A). In the future, more investigation should be performed to uncover the veil between these two cell types. As for the detail mechanism, how *TNMD* promotes cardiac fibrosis of ARVC need more investigation in the future.

The limitation of our study included three points: the small sample size of healthy controls (*n* = 2), the enrollment of ARVC patients with end-stage heart failure patients, and the other was lack of in-depth mechanisms. First, due to the difficulty to get heart tissue, especially fresh and healthy heart tissue, we only collected 24 samples from 8 objects (only 2 for healthy controls) in total. However, such sample size was accepted in the area of scRNA-seq for healthy human myocardium [12, 60]. What is more, scRNA-seq paid more attention to the cell number than patients’ sample size, and we enrolled 247,684 cells (including 198,927 cells from patients with ARVC) for analysis, which was hug cell number compared to previous scRNA-seq studies for ARVC, no matter in animals [43] or in human [16]. In addition, key findings from scRNA-seq data were further validated by pathological staining in more human samples or by animal experiments to sure robust science. Second, the ARVC patients enrolled in the present study were end-stage heart failure patients, meaning that the cellular landscape in ARVC might be affected by heart failure. However, it does not affect the reliability of the key conclusions in the study. Third, as for in-depth mechanisms, we have identified some key genes involved with the pathogenesis of immune cells (*NLRP3* for macrophages) and fibroblasts (*TNMD*). Although we have investigated that targeting on NLRP3 with MCC950 could significantly alleviate the cardiac phenotype in a ARVC mouse model, more mechanism study should be performed to apply in clinical practice. Especially for cellular source of the adipocytes and the signals inducing the formation of adipocytes, genetic lineage tracing approaches in pig models of ARVC ought to be useful in addressing this question, because of that mouse models of ARVC generally do not have the robust fatty involvement seen in humans [61, 62].

## Conclusions

We provided a comprehensive analysis of the lineage-specific changes in the blood and cardiac tissues from ARVC patients at a single-cell resolution. We found that *NLRP3* contributed into the pathogenesis of ARVC via regulating the myeloid cells, especially the CCL3^+^ macrophages. Pharmacological inhibition of NLRP3 could serve as a promising medical therapy for an early stage of ARVC which was characteristic with right ventricle dilation and dysfunction. We also identified fibroblasts as a potential cellular source of adipocytes.

### Supplementary Information

**Additional file 1: Table S1.** Clinical information of ARVC patients based on Task Force Criteria in 2010. **Table S2.** Clinical characteristics of enrolled ARVC patients and normal controls. **Table S3.** Counts of different biotypes. **Table S4.** Cell types assignment by using SingleR and manual annotation. **Table S5.** Current list of GWAS cardiac arrhythmia genes. **Table S6.** The summary of major non-cardiomyocytes subpopulations in ARVC and normal human hearts.

**Additional file 2: Fig. S1.** Quality control and preliminary scRNA-seq data analysis. **Fig. S2.** Cell type identification. **Fig. S3.** Cell type confirmation. **Fig. S4.** Myeloid subpopulations. **Fig. S5.** The expression of inflammatory genes and trajectory analysis of myeloid. **Fig. S6.** Fibroblast subpopulations. **Fig. S7.** Predicted and altered cell-cell interactions in ARVC patient hearts. **Fig. S8.** Predicted and altered Mye2-cell interactions in ARVC patient hearts. **Fig. S9.** NLRP3 in ARVC patient hearts. **Fig. S10.** Pharmacological inhibition of NLRP3 significantly alleviate the fibrosis and inflammation in ARVC mouse.

**Additional file 3.** The ARRIVE guidelines 2.0: author checklist.

**Additional file 4.** Major cluster DEGs.

**Additional file 5.** Myeloid cluster DEGs.

**Additional file 6.** Fibroblast cluster DEGs.

**Additional file 7.** Group compare in major cluster DEGs.

## Data Availability

The datasets generated during the current study are available at Genome Sequence Archive (accession no. HRA005871).

## Abbreviations

AC_LV: Left ventricle of arrhythmogenic right ventricular cardiomyopathy
AC_PBMC: Peripheral blood mononuclear cell of arrhythmogenic right ventricular cardiomyopathy
AC_RV: Right ventricle of arrhythmogenic right ventricular cardiomyopathy
ARVC: Arrhythmogenic right ventricular cardiomyopathy
DC: Dendritic cell
DEGs: Differentially expressed genes
DMEM: Dulbecco’s modified Eagle’s medium
DSG2: Desmoglein 2
EC: Endothelial cell
ECG: Electrocardiogram
FB: Fibroblast
FBS: Fetal bovine serum
GEM: Gel bead in emulsion
GO: Gene ontology
GWAS: Genome-wide association study
HBSS: Hanks’ Balanced Salt Solution
NC: Normal controls
NC_LV: Left ventricle of normal control
NC_RV: Right ventricle of normal control
NCM: Non-cardiomyocytes
NK: Natural killer
NLRP3: NOD-like receptor protein 3
PBMC: Peripheral blood mononuclear cell
PBS: Phosphate buffered saline
PCR: Polymerase chain reaction
scRNA-seq: Single-cell RNA sequencing
TF: Transcription factor
UMAP: Uniform manifold approximation and projection
VSMC: Vascular smooth muscle cell
WB: Washing buffer
WT: Wild type

## References

[1] Leren IS, Saberniak J, Haland TF, Edvardsen T, Haugaa KH (2017). Combination of ECG and echocardiography for identification of arrhythmic events in early ARVC. JACC Cardiovasc Imaging.

[2] Chen S, Chen L, Saguner AM, Chen K, Akdis D, Gasperetti A, Brunckhorst C, Tang H, Guo G, Rao M (2022). Novel risk prediction model to determine adverse heart failure outcomes in arrhythmogenic right ventricular cardiomyopathy. J Am Heart Assoc.

[3] Chelko SP, Asimaki A, Lowenthal J, Bueno-Beti C, Bedja D, Scalco A, Amat-Alarcon N, Andersen P, Judge DP, Tung L (2019). Therapeutic modulation of the immune response in arrhythmogenic cardiomyopathy. Circulation.

[4] Campuzano O, Alcalde M, Iglesias A, Barahona-Dussault C, Sarquella-Brugada G, Benito B, Arzamendi D, Flores J, Leung TK, Talajic M (2012). Arrhythmogenic right ventricular cardiomyopathy: severe structural alterations are associated with inflammation. J Clin Pathol.

[5] Moccia F, Lodola F, Stadiotti I, Pilato CA, Bellin M, Carugo S, Pompilio G, Sommariva E, Maione AS (2019). Calcium as a Key Player in Arrhythmogenic Cardiomyopathy: Adhesion Disorder or Intracellular Alteration?. Int J Mol Sci.

[6] Asatryan B, Asimaki A, Landstrom AP, Khanji MY, Odening KE, Cooper LT, Marchlinski FE, Gelzer AR, Semsarian C, Reichlin T (2021). Inflammation and immune response in arrhythmogenic cardiomyopathy: state-of-the-art review. Circulation.

[7] Chen L, Song J, Chen X, Chen K, Ren J, Zhang N, Rao M, Hu Z, Zhang Y, Gu M (2019). A novel genotype-based clinicopathology classification of arrhythmogenic cardiomyopathy provides novel insights into disease progression. Eur Heart J.

[8] Shu S, Fu M, Chen X, Zhang N, Zhao R, Chang Y, Cui H, Liu Z, Wang X, Hua X, et al. Cellular landscapes of nondiseased human cardiac valves from end-stage heart failure-explanted heart. Arterioscler Thromb Vasc Biol. 2022:42(12):1429–46. 10.1161/ATVBAHA.122.318314 36200446

[9] Li Y, Ren P, Dawson A, Vasquez HG, Ageedi W, Zhang C, Luo W, Chen R, Li Y, Kim S (2020). Single-cell transcriptome analysis reveals dynamic cell populations and differential gene expression patterns in control and aneurysmal human aortic tissue. Circulation.

[10] Krijgsman D, de Vries NL, Skovbo A, Andersen MN, Swets M, Bastiaannet E, Vahrmeijer AL, van de Velde CJH, Heemskerk MHM, Hokland M (2019). Characterization of circulating T-, NK-, and NKT cell subsets in patients with colorectal cancer: the peripheral blood immune cell profile. Cancer Immunol Immunother.

[11] Kim N, Kim HK, Lee K, Hong Y, Cho JH, Choi JW, Lee JI, Suh YL, Ku BM, Eum HH (2020). Single-cell RNA sequencing demonstrates the molecular and cellular reprogramming of metastatic lung adenocarcinoma. Nat Commun.

[12] Rao M, Wang X, Guo G, Wang L, Chen S, Yin P, Chen K, Chen L, Zhang Z, Chen X (2021). Resolving the intertwining of inflammation and fibrosis in human heart failure at single-cell level. Basic Res Cardiol.

[13] Vijay J, Gauthier MF, Biswell RL, Louiselle DA, Johnston JJ, Cheung WA, Belden B, Pramatarova A, Biertho L, Gibson M (2020). Single-cell analysis of human adipose tissue identifies depot and disease specific cell types. Nat Metab.

[14] Zhao G, Lu H, Chang Z, Zhao Y, Zhu T, Chang L, Guo Y, Garcia-Barrio MT, Chen YE, Zhang J. Single cell RNA sequencing reveals the cellular heterogeneity of aneurysmal infrarenal abdominal aorta. Cardiovasc Res. 2021;117(5):1402–16. 10.1093/cvr/cvaa214 PMC8064434 32678909

[15] Sárvári AK, Van Hauwaert EL, Markussen LK, Gammelmark E, Marcher AB, Ebbesen MF, Nielsen R, Brewer JR, Madsen JGS, Mandrup S (2021). Plasticity of epididymal adipose tissue in response to diet-induced obesity at single-nucleus resolution. Cell Metab.

[16] Reichart D, Lindberg EL, Maatz H, Miranda AMA, Viveiros A, Shvetsov N, Gärtner A, Nadelmann ER, Lee M, Kanemaru K (2022). Pathogenic variants damage cell composition and single cell transcription in cardiomyopathies. Science.

[17] Cheng S, Li Z, Gao R, Xing B, Gao Y, Yang Y, Qin S, Zhang L, Ouyang H, Du P (2021). A pan-cancer single-cell transcriptional atlas of tumor infiltrating myeloid cells. Cell.

[18] Kowalczyk MS, Tirosh I, Heckl D, Rao TN, Dixit A, Haas BJ, Schneider RK, Wagers AJ, Ebert BL, Regev A (2015). Single-cell RNA-seq reveals changes in cell cycle and differentiation programs upon aging of hematopoietic stem cells. Genome Res.

[19] Street K, Risso D, Fletcher RB, Das D, Ngai J, Yosef N, Purdom E, Dudoit S (2018). Slingshot: cell lineage and pseudotime inference for single-cell transcriptomics. BMC Genomics.

[20] Jin S, Guerrero-Juarez CF, Zhang L, Chang I, Ramos R, Kuan CH, Myung P, Plikus MV, Nie Q (2021). Inference and analysis of cell-cell communication using Cell Chat. Nat Commun.

[21] Auton A, Brooks LD, Durbin RM, Garrison EP, Kang HM, Korbel JO, Marchini JL, McCarthy S, McVean GA, Genomes Project C (2015). A global reference for human genetic variation. Nature.

[22] Lek M, Karczewski KJ, Minikel EV, Samocha KE, Banks E, Fennell T, O’Donnell-Luria AH, Ware JS, Hill AJ, Cummings BB (2016). Analysis of protein-coding genetic variation in 60,706 humans. Nature.

[23] Kumar P, Henikoff S, Ng PC (2009). Predicting the effects of coding non-synonymous variants on protein function using the SIFT algorithm. Nat Protoc.

[24] Adzhubei IA, Schmidt S, Peshkin L, Ramensky VE, Gerasimova A, Bork P, Kondrashov AS, Sunyaev SR (2010). A method and server for predicting damaging missense mutations. Nat Methods.

[25] Schwarz JM, Rodelsperger C, Schuelke M, Seelow D (2010). MutationTaster evaluates disease-causing potential of sequence alterations. Nat Methods.

[26] Kircher M, Witten DM, Jain P, O’Roak BJ, Cooper GM, Shendure J (2014). A general framework for estimating the relative pathogenicity of human genetic variants. Nat Genet.

[27] Muona M, Berkovic SF, Dibbens LM, Oliver KL, Maljevic S, Bayly MA, Joensuu T, Canafoglia L, Franceschetti S, Michelucci R (2015). A recurrent de novo mutation in KCNC1 causes progressive myoclonus epilepsy. Nat Genet.

[28] Richards S, Aziz N, Bale S, Bick D, Das S, Gastier-Foster J, Grody WW, Hegde M, Lyon E, Spector E (2015). Standards and guidelines for the interpretation of sequence variants: a joint consensus recommendation of the American College of Medical Genetics and Genomics and the Association for Molecular Pathology. Genet Med.

[29] Fu M, Xu L, Chen X, Han W, Ruan C, Li J, Cai C, Ye M, Gao P (2019). Neural crest cells differentiate into brown adipocytes and contribute to periaortic arch adipose tissue formation. Arterioscler Thromb Vasc Biol.

[30] Percie du Sert N, Hurst V, Ahluwalia A, Alam S, Avey MT, Baker M, Browne WJ, Clark A, Cuthill IC, Dirnagl U (2020). The ARRIVE guidelines 2.0: updated guidelines for reporting animal research. PLoS Biol.

[31] Lin YN, Mesquita T, Sanchez L, Chen YH, Liu W, Li C, Rogers R, Wang Y, Li X, Wu D (2021). Extracellular vesicles from immortalized cardiosphere-derived cells attenuate arrhythmogenic cardiomyopathy in desmoglein-2 mutant mice. Eur Heart J.

[32] Coll RC, Robertson AA, Chae JJ, Higgins SC, Muñoz-Planillo R, Inserra MC, Vetter I, Dungan LS, Monks BG, Stutz A (2015). A small-molecule inhibitor of the NLRP3 inflammasome for the treatment of inflammatory diseases. Nat Med.

[33] Syed F, Diwan A, Hahn HS (2005). Murine echocardiography: a practical approach for phenotyping genetically manipulated and surgically modeled mice. J Am Soc Echocardiogr.

[34] Guo Y, Cao Y, Jardin BD, Zhang X, Zhou P, Guatimosim S, Lin J, Chen Z, Zhang Y, Mazumdar N, et al. Ryanodine receptor 2 (RYR2) dysfunction activates the unfolded protein response and perturbs cardiomyocyte maturation. Cardiovasc Res. 2023;119(1):221–35. 10.1093/cvr/cvac077 PMC10233305 35576474

[35] Skelly DA, Squiers GT, McLellan MA, Bolisetty MT, Robson P, Rosenthal NA, Pinto AR (2018). Single-cell transcriptional profiling reveals cellular diversity and intercommunication in the mouse heart. Cell Rep.

[36] Wang L, Yu P, Zhou B, Song J, Li Z, Zhang M, Guo G, Wang Y, Chen X, Han L (2020). Single-cell reconstruction of the adult human heart during heart failure and recovery reveals the cellular landscape underlying cardiac function. Nat Cell Biol.

[37] Chaffin M, Papangeli I, Simonson B, Akkad A-D, Hill MC, Arduini A, Fleming SJ, Melanson M, Hayat S, Kost-Alimova M (2022). Single-nucleus profiling of human dilated and hypertrophic cardiomyopathy. Nature.

[38] Basso C, Thiene G, Corrado D, Angelini A, Nava A, Valente M (1996). Arrhythmogenic right ventricular cardiomyopathy. Dysplasia, dystrophy, or myocarditis?. Circulation.

[39] Buniello A, MacArthur JAL, Cerezo M, Harris LW, Hayhurst J, Malangone C, McMahon A, Morales J, Mountjoy E, Sollis E (2019). The NHGRI-EBI GWAS Catalog of published genome-wide association studies, targeted arrays and summary statistics 2019. Nucleic Acids Res.

[40] Weckbach LT, Grabmaier U, Uhl A, Gess S, Boehm F, Zehrer A, Pick R, Salvermoser M, Czermak T, Pircher J (2019). Midkine drives cardiac inflammation by promoting neutrophil trafficking and NETosis in myocarditis. J Exp Med.

[41] Pellegrini C, Martelli A, Antonioli L, Fornai M, Blandizzi C, Calderone V (2021). NLRP3 inflammasome in cardiovascular diseases: Pathophysiological and pharmacological implications. Med Res Rev.

[42] Chelko SP, Asimaki A, Andersen P, Bedja D, Amat-Alarcon N, DeMazumder D, Jasti R, MacRae CA, Leber R, Kleber AG (2016). Central role for GSK3β in the pathogenesis of arrhythmogenic cardiomyopathy. JCI Insight.

[43] Yuan P, Cheedipudi SM, Rouhi L, Fan S, Simon L, Zhao Z, Hong K, Gurha P, Marian AJ (2021). Single-cell RNA sequencing uncovers paracrine functions of the epicardial-derived cells in arrhythmogenic cardiomyopathy. Circulation.

[44] Kohela A, van Kampen SJ, Moens T, Wehrens M, Molenaar B, Boogerd CJ, Monshouwer-Kloots J, Perini I, Goumans MJ, Smits AM (2021). Epicardial differentiation drives fibro-fatty remodeling in arrhythmogenic cardiomyopathy. Sci Transl Med.

[45] Lin YN, Ibrahim A, Marbán E, Cingolani E (2021). Pathogenesis of arrhythmogenic cardiomyopathy: role of inflammation. Basic Res Cardiol.

[46] Lubos N, van der Gaag S, Gerçek M, Kant S, Leube RE, Krusche CA (2020). Inflammation shapes pathogenesis of murine arrhythmogenic cardiomyopathy. Basic Res Cardiol.

[47] Gruber CN, Patel RS, Trachtman R, Lepow L, Amanat F, Krammer F, Wilson KM, Onel K, Geanon D, Tuballes K (2020). Mapping systemic inflammation and antibody responses in multisystem inflammatory syndrome in children (MIS-C). Cell.

[48] Mangan MSJ, Olhava EJ, Roush WR, Seidel HM, Glick GD, Latz E (2018). Targeting the NLRP3 inflammasome in inflammatory diseases. Nat Rev Drug Discov.

[49] Shi K, Li H, Chang T, He W, Kong Y, Qi C, Li R, Huang H, Zhu Z, Zheng P (2022). Bone marrow hematopoiesis drives multiple sclerosis progression. Cell.

[50] Grant AJ, Yang N, Moore MJ, Lam YT, Michael PL, Chan AHP, Santos M, Rnjak-Kovacina J, Tan RP, Wise SG (2023). Selective NLRP3 inflammasome inhibitor MCC950 suppresses inflammation and facilitates healing in vascular materials. Adv Sci (Weinh).

[51] Minns MS, Liboro K, Lima TS, Abbondante S, Miller BA, Marshall ME, Tran Chau J, Roistacher A, Rietsch A, Dubyak GR (2023). NLRP3 selectively drives IL-1beta secretion by Pseudomonas aeruginosa infected neutrophils and regulates corneal disease severity. Nat Commun.

[52] Austin KM, Trembley MA, Chandler SF, Sanders SP, Saffitz JE, Abrams DJ, Pu WT (2019). Molecular mechanisms of arrhythmogenic cardiomyopathy. Nat Rev Cardiol.

[53] Leyva F, Zegard A, Okafor O, Foley P, Umar F, Taylor RJ, Marshall H, Stegemann B, Moody W, Steeds RP (2022). Myocardial fibrosis predicts ventricular arrhythmias and sudden death after cardiac electronic device implantation. J Am Coll Cardiol.

[54] Moon SJ, Bae JM, Park KS, Tagkopoulos I, Kim KJ (2019). Compendium of skin molecular signatures identifies key pathological features associated with fibrosis in systemic sclerosis. Ann Rheum Dis.

[55] Unamuno X, Gomez-Ambrosi J, Ramirez B, Rodriguez A, Becerril S, Valenti V, Moncada R, Silva C, Salvador J, Fruhbeck G (2020). Dermatopontin, A novel adipokine promoting adipose tissue extracellular matrix remodelling and inflammation in obesity. J Clin Med.

[56] You Y, Yuan H, Min H, Li C, Chen J (2023). Fibroblast-derived CXCL14 aggravates crystalline silica-induced pulmonary fibrosis by mediating polarization and recruitment of interstitial macrophages. J Hazard Mater.

[57] van Kuijk K, Demandt JAF, Perales-Paton J, Theelen TL, Kuppe C, Marsch E, de Bruijn J, Jin H, Gijbels MJ, Matic L (2022). Deficiency of myeloid PHD proteins aggravates atherogenesis via macrophage apoptosis and paracrine fibrotic signalling. Cardiovasc Res.

[58] Cheng J, Wu H, Xie C, He Y, Mou R, Zhang H, Yang Y, Xu Q. Single Cell mapping of large and small arteries during hypertensive aging. J Gerontol A Biol Sci Med Sci. 2023:glad188. 10.1093/gerona/glad188 37531301

[59] Lim GB (2019). Midkine promotes NETosis in myocarditis. Nat Rev Cardiol.

[60] Liu X, Yin K, Chen L, Chen W, Li W, Zhang T, Sun Y, Yuan M, Wang H, Song Y (2023). Lineage-specific regulatory changes in hypertrophic cardiomyopathy unraveled by single-nucleus RNA-seq and spatial transcriptomics. Cell Discov.

[61] Garcia-Gras E, Lombardi R, Giocondo MJ, Willerson JT, Schneider MD, Khoury DS, Marian AJ (2006). Suppression of canonical Wnt/beta-catenin signaling by nuclear plakoglobin recapitulates phenotype of arrhythmogenic right ventricular cardiomyopathy. J Clin Invest.

[62] Yang Z, Bowles NE, Scherer SE, Taylor MD, Kearney DL, Ge S, Nadvoretskiy VV, DeFreitas G, Carabello B, Brandon LI (2006). Desmosomal dysfunction due to mutations in desmoplakin causes arrhythmogenic right ventricular dysplasia/cardiomyopathy. Circ Res.

