# Supplementary material for: Single-cell RNA sequencing in donor and end-stage heart failure patients identifies NLRP3 as a therapeutic target for arrhythmogenic right ventricular cardiomyopathy
Source: BMC Med. 2024 Jan 8;22:11. doi: 10.1186/s12916-023-03232-8 (PMC10773142; doi:10.1186/s12916-023-03232-8)
Supplement: Supplementary file 1 — Additional file 1: Table S1. Clinical information of ARVC patients based on Task Force Criteria in 2010. Table S2. Clinical characteristics of enrolled ARVC patients and normal controls. Table S3. Counts of different biotypes. Table S4. Cell types assignment by using SingleR and manual annotation. Table S5. Current list of GWAS cardiac arrhythmia genes. Table S6. The summary of major non-cardiomyocytes subpopulations in ARVC and normal human hearts. [file 12916_2023_3232_MOESM1_ESM.docx]

**Additional file 1**

**Table S1. Clinical information of ARVC patients based on Task Force Criteria in 2010.**

| ID | Gene mutation | pathology | | Repolarization abnormalities | | Depolarization/conduction abnormalities | | Arrhythmias | | Family history* | | Structural alterations | | Diagnosis |
| --- | --- | --- | --- | --- | --- | --- | --- | --- | --- | --- | --- | --- | --- | --- |
|  | **major** | **major** | **minor** | **major** | **minor** | **major** | **minor** | **major** | **minor** | **Major** | **minor** | **major** | **minor** | **Major/minor** |
| ARVC_1 | DSG2 | 1 |  | 1 |  | 1 |  |  |  |  |  | 1 |  | 4/0 |
| ARVC_2 | PKP2 | 1 |  | 1 |  | 1 |  |  |  |  |  | 1 |  | 4/0 |
| ARVC_3 | None | 1 |  | 1 |  | 1 |  |  | 1 |  |  | 1 |  | 4/1 |
| ARVC_4 | DSC2 | 1 |  | 1 |  | 1 |  |  | 1 |  |  | 1 |  | 4/1 |
| ARVC_5 | None | 1 |  | 1 |  | 1 |  |  |  |  |  | 1 |  | 4/0 |
| ARVC_6 | None | 1 |  | 1 |  | 1 |  |  |  |  |  | 1 |  | 4/0 |

ARVC, arrhythmogenic right ventricular cardiomyopathy.

**NOTE**

*: The family history does not include gene mutation information.

**Table S2. Clinical characteristics of enrolled ARVC patients and normal controls.**

|  | **NC-1** | **NC-2** | **ARVC-1** | **ARVC-2** | **ARVC-3** | **ARVC-4** | **ARVC-5** | **ARVC-6** |
| --- | --- | --- | --- | --- | --- | --- | --- | --- |
| Sex | male | male | male | male | male | male | male | male |
| Age | 50 | 53 | 35 | 45 | 61 | 37 | 64 | 39 |
| Palpitation | NA | NA | 1 | 0 | 1 | 0 | 0 | 1 |
| Chest distress | NA | NA | 1 | 1 | 1 | 1 | 1 | 0 |
| Dyspnea | NA | NA | 0 | 0 | 1 | 1 | 0 | 0 |
| ACEI/ARB | NA | NA | 0 | 1 | 0 | 1 | 1 | 0 |
| β-blocker | NA | NA | 1 | 1 | 1 | 1 | 1 | 1 |
| Hypertension | NA | NA | 0 | 0 | 0 | 0 | 0 | 0 |
| Hyperlipidemia | NA | NA | 0 | 0 | 0 | 0 | 0 | 0 |
| Diabetes | NA | NA | 0 | 0 | 1 | 0 | 1 | 0 |
| ICD | NA | NA | 0 | 0 | 1 | 0 | 0 | 0 |
| NYHA class | NA | NA | Ⅲ | Ⅳ | Ⅲ | Ⅲ | Ⅳ | Ⅲ |
| Weight(kg) | NA | NA | 72.0 | 47.5 | 56.6 | 82.0 | 73.0 | 68.6 |
| Height(m) | NA | NA | 1.75 | 1.62 | 1.60 | 1.82 | 1.70 | 1.68 |
| Aortic dimension(mm) | NA | NA | 25 | 26 | 25 | 26 | 36 | 24 |
| LA diameter (mm) | NA | NA | 19 | 28 | 37 | 36 | 44 | 34 |
| LVEDD (mm) | NA | NA | 35 | 35 | 48 | 61 | 58 | 61 |
| IVS (mm) | NA | NA | 9 | 8 | 10 | 10 | 8 | 9 |
| RVEDD (mm) | NA | NA | 47 | 57 | 34 | 44 | 40 | 39 |
| LVEF (%) | 65 | 60 | 53 | 50 | 35 | 35 | 32 | 26 |
| Ventricular assist device | NA | NA | NO | NO | NO | NO | NO | NO |
| Chronic inotropic therapy | NA | NA | NO | NO | NO | NO | NO | NO |

ARVC, arrhythmogenic right ventricular cardiomyopathy; NC, normal control; ICD, implantable cardioverter-defibrillator; NYHA, New York Heart Association; LA, left atria; LVEDD, left ventricular end-diastolic diameter; IVS, interventricular septum; RVEDD, right ventricular end-diastolic diameter; LVEF, left ventricular ejection fraction.

**Table S3. Counts of different biotypes.**

| **Biotype** | **n** |
| --- | --- |
| IG_C_gene | 14 |
| IG_C_pseudogene | 9 |
| IG_D_gene | 37 |
| IG_J_gene | 18 |
| IG_J_pseudogene | 3 |
| IG_V_gene | 144 |
| IG_V_pseudogene | 188 |
| lncRNA | 16562 |
| protein_coding | 19394 |
| TR_C_gene | 6 |
| TR_D_gene | 4 |
| TR_J_gene | 79 |
| TR_J_pseudogene | 4 |
| TR_V_gene | 106 |
| TR_V_pseudogene | 33 |

**Table S4. Cell types assignment by using SingleR and manual annotation.**

| **Clusters** | **SingleR** | **Manual** | **Markers** | **References** |
| --- | --- | --- | --- | --- |
| 0 | T_cells | T cells | CD3D, CD3E | [9-11] |
| 1 | Monocyte | Myeloid | LYZ, CD68, S100A8, CXCR2, CD74 | [9, 12-14] |
| 2 | Endothelial_cells | Endothelial cells | VWF, PECAM | [9, 12, 14] |
| 3 | T_cells | T cells | CD3D, CD3E | [9-11] |
| 4 | NK_cell | NK cells | NKG7, GNLY, GZMA | [9-11] |
| 5 | NK_cell | NK cells | NKG7, GNLY, GZMA | [9-11] |
| 6 | Fibroblast | Fibroblast | DCN, COL1A1 | [15] |
| 7 | Endothelial_cells | Endothelial cells | VWF, PECAM | [9, 12, 14] |
| 8 | Macrophage | Myeloid | LYZ, CD68, S100A8, CXCR2, CD74 | [9, 12-14] |
| 9 | Monocyte | Myeloid | LYZ, CD68, S100A8, CXCR2, CD74 | [9, 12-14] |
| 10 | NK_cell | NK cells | NKG7, GNLY, GZMA | [9-11] |
| **11** | **Smooth_muscle_cells** | **Perictyte** | **ABCC9, RGS5** | **[12]** |
| 12 | Smooth_muscle_cells | VSMC | ACTA2. MYH11 | [9, 14] |
| 13 | Endothelial_cells | Endothelial cells | VWF, PECAM | [9, 12, 14] |
| 14 | Monocyte | Myeloid | LYZ, CD68, S100A8, CXCR2, CD74 | [9, 12-14] |
| 15 | B_cells | B cells | MS4A1, CD79A | [12] |
| 16 | Endothelial_cells | Endothelial cells | VWF, PECAM | [9, 12, 14] |
| 17 | T_cells | T cells | CD3D, CD3E | [9-11] |
| 18 | Monocyte | Myeloid | LYZ, CD68, S100A8, CXCR2, CD74 | [9, 12-14] |
| 19 | Endothelial_cells | Endothelial cells | VWF, PECAM | [9, 12, 14] |
| 20 | Neutrophils | Neutrophils | CSF3R, FCGR3B | [9, 12-14] |
| **21** | **Smooth_muscle_cells** | **Cardiomycyte** | **ACTC1, MYL2** | **[12]** |
| 22 | Monocyte | Myeloid | LYZ, CD68, S100A8, CXCR2, CD74 | [9, 12-14] |
| 23 | Smooth_muscle_cells | VSMC | ACTA2. MYH11 | [9, 14] |
| 24 | Macrophage | Myeloid | LYZ, CD68, S100A8, CXCR2, CD74 | [9, 12-14] |
| 25 | Fibroblast | Fibroblast | DCN, COL1A1 | [15] |
| 26 | Endothelial_cells | Endothelial cells | VWF, PECAM | [9, 12, 14] |
| 27 | Macrophage | Myeloid | LYZ, CD68, S100A8, CXCR2, CD74 | [9, 12-14] |
| 28 | Mast_cells | Mast cells | TPSAB1, TPSB2 | [9] |
| 29 | Endothelial_cells | Endothelial cells | VWF, PECAM | [9, 12, 14] |
| **30** | **Monocyte** | **Plasma cells** | **MZB1, IGHG1** | **[9]** |
| 31 | T_cells | T cells | CD3D, CD3E | [9-11] |
| **32** | **Endothelial_cells** | **Doublets** | **PECAM, ACTA2, MYL2** |  |
| 33 | Undefined | Low-quality |  |  |
| 34 | Neuron | Neuron | PLP1, NRXN1 | [16] |
| **35** | **Macrophage** | **Doublets** | **CD68, JCHAIN, PECAM** |  |
| 36 | Undefined | Low-quality |  |  |

**Table S5. Current list of GWAS cardiac arrhythmia genes.**

| **DISEASE.TRAIT** | **SNP_SYMBOL_IDS** |
| --- | --- |
| Sudden cardiac arrest | AP1G2 |
| Sudden cardiac arrest | PARP4 |
| Sudden cardiac arrest | ATF1 |
| Sudden cardiac arrest | PLCE1 |
| Sudden cardiac arrest | GRIA1 |
| Sudden cardiac arrest | TMEFF2 |
| Sudden cardiac arrest | RSPH6A |
| Sudden cardiac arrest | ACYP2 |
| Sudden cardiac arrest | ZNF385B |
| Sudden cardiac arrest | KCTD1 |
| Sudden cardiac arrest | CHRNB4 |
| Sudden cardiac arrest | CDH4 |
| Sudden cardiac arrest | DEGS2 |
| Sudden cardiac arrest | CLSTN2 |
| Sudden cardiac arrest | CHL1 |
| Sudden cardiac arrest | ESR1 |
| Sudden cardiac arrest | RAP1GAP2 |
| Sudden cardiac arrest | SIN3A |
| Supraventricular ectopy | SCN5A |
| Supraventricular ectopy | KIF6 |
| Ventricular ectopy | MAP3K7CL |
| Ventricular ectopy | FAF1 |
| Sudden cardiac arrest | RAB3GAP1 |
| Sudden cardiac arrest | ZRANB3 |
| Sudden cardiac arrest | ZNF365 |
| Sudden cardiac arrest | BAZ2B |
| Sudden cardiac arrest | NGEF |
| Sudden cardiac arrest | ZFPM2 |

**Table S6. The summary of major non-cardiomyocytes subpopulations in ARVC and normal human hearts.**

| **Cell cluster (Annotation)** | **Cell number** | **Marker genes** | **Transcription factors** | **Major function** | **Major position** |
| --- | --- | --- | --- | --- | --- |
| Mye0 (MHC^low^ M2-like MP) | 10239 | SELENOP, RNASE1, MRC1 | MAF, TCF4 | Receptor-mediated endocytosis |  |
| Mye1 (Classic MO) | 7335 | S100A8, S100A9, VCAN | FOXP1, KLF5 | Neutrophil activation | AC_PBMC |
| Mye2 (M1-like MP) | 5311 | CCL3, CCL4, IL1B | NLRP3, NFKB2 | Pro-inflammation | AC_RV |
| Mye3 (Nonclassic MO) | 4918 | FCN1, FCGR3A, SPN | POU2F2, SMAD1 | Regulation of lymphocyte proliferation | AC_LV/RV |
| Mye4 (MO-derived MP) | 4662 | CD14, S100A9, CD68 | BACH1, ZNF148 | Leukocyte migration |  |
| Mye5 (cDC2) | 4432 | CD1C, HLA-DQB1, FCER1A | ZNF677, TGIF1 | Antigen processing and presentation |  |
| Mye6 (TREM2^+^ MP) | 4202 | CD68, TREM2, FABP5 | ENO1, THRA | Response to lipoprotein particle |  |
| Mye7 (HSP^+^ MP) | 3642 | MERTK, HSPD1, HSPB1 | ZFHX3, KLF6 | Protein folding |  |
| Mye8 (MHC^intermediate^ M2-like) | 3478 | CD14, CD68, HLA-DQB1 | MAFB, EGR2 | Antigen processing and presentation |  |
| Mye9 (MO-derived DC) | 2877 | EREG, VCAN, ITGAX | GABPB1, BCL3 | Leukocyte migration |  |
| Mye10 (MYL2^+^ MP2) | 2545 | MYL2, TPM1, MERTK | DDIT3, KLF9 | Muscle contraction |  |
| Mye11 (Proliferative MP) | 719 | HIST1H4C, STMN1, CD68 | ENO1, HIST1H2BN | Proliferative macrophage | NC_LV |
| Mye12 (cDC1) | 450 | HLA-DQB1, IDO1, CCSER1 | HDX, ZEB1 | Antigen processing and presentation |  |
| Mye13 (LAMP3^+^ DC) | 136 | LAMP3, HLA-DQB1, CD1C | NFE2L1, ZFP62 | Antigen processing and presentation |  |
| FB0 (FB_activated) | 5019 | POSTN, THBS4, HSPA1A | HEY1, SOX15 | Response to TGFb, ECM organization | AC_LV/RV |
| FB1 (FB_silent) | 3957 | PLA2G2A, CCL2, FGF7 | ZNF35, WT1 | ECM organization |  |
| FB2 (FB_SCN7A) | 2435 | SCN7A, LAMB1, ABCA8 | RUNX2, ZNF16 |  |  |
| FB3 (FAP_like) | 2428 | PTGDS, CFD, PDGFRA | PPARG, CEBPD | Adipogenic potential | AC_RV |
| FB4 (FB_stromal) | 1413 | MFAP5, IGFBP6, CD34 | CD59, NONO | Stromal fibroblast |  |
| FB5 (myoFB) | 852 | ACTA2, TAGLN, MYL9 | MECOM, FOXC2 | Muscle contraction, ECM organization | AC_RV |
| FB6 (FB_FABP4) | 527 | FABP4, FABP5, CD36 | PPARG, ETS1 | Endothelial differentiation | AC_LV/RV |

ARVC, arrhythmogenic right ventricular cardiomyopathy; NC, normal control; AC_LV, left ventricle of arrhythmogenic right ventricular cardiomyopathy; AC_RV, right ventricle of arrhythmogenic right ventricular cardiomyopathy; NC_LV, left ventricle of normal control; NC_RV, right ventricle of normal control; ; MP, macrophage; MO, monocyte; DC, dendritic cell.
