# Supplementary material for: Single-cell RNA sequencing in donor and end-stage heart failure patients identifies NLRP3 as a therapeutic target for arrhythmogenic right ventricular cardiomyopathy
Source: BMC Med. 2024 Jan 8;22:11. doi: 10.1186/s12916-023-03232-8 (PMC10773142; doi:10.1186/s12916-023-03232-8)
Supplement: Supplementary file 2 — Additional file 2: Fig. S1. Quality control and preliminary scRNA-seq data analysis. Fig. S2. Cell type identification. Fig. S3. Cell type confirmation. Fig. S4. Myeloid subpopulations. Fig. S5. The expression of inflammatory genes and trajectory analysis of myeloid. Fig. S6. Fibroblast subpopulations. Fig. S7. Predicted and altered cell-cell interactions in ARVC patient hearts. Fig. S8. Predicted and altered Mye2-cell interactions in ARVC patient hearts. Fig. S9. NLRP3 in ARVC patient hearts. Fig. S10. Pharmacological inhibition of NLRP3 significantly alleviate the fibrosis and inflammation in ARVC mouse. [file 12916_2023_3232_MOESM2_ESM.docx]

**Additional file 2**





**Fig. S1. Quality control and preliminary scRNA-seq data analysis. A–B**, Count_RNA number, Feature_RNA number, mitochondrial and hemoglobin RNA percentage of cells in 40 samples before (**A**) and after (**B**) quality control. **C-D**, UMAP plots of the 252,269 cells colored by 37 clusters (**C**) or 40 samples (**D**). The batch effect among the different samples was corrected using the Seurat V4 integration algorithm. We combined SingleR with manual annotation to assign cell types to the different clusters. The clustering analysis yielded 37 clusters (C0–C36). UMAP, uniform manifold approximation and projection; ARVC, arrhythmogenic right ventricular cardiomyopathy; NC, normal control; AC_LV, ARVC left ventricle; AC_RV, ARVC right ventricle; AC_PBMC, ARVC PBMC; NC_LV, NC left ventricle; NC_RV, NC right ventricle; PBMC peripherial blood mononuclear cell.


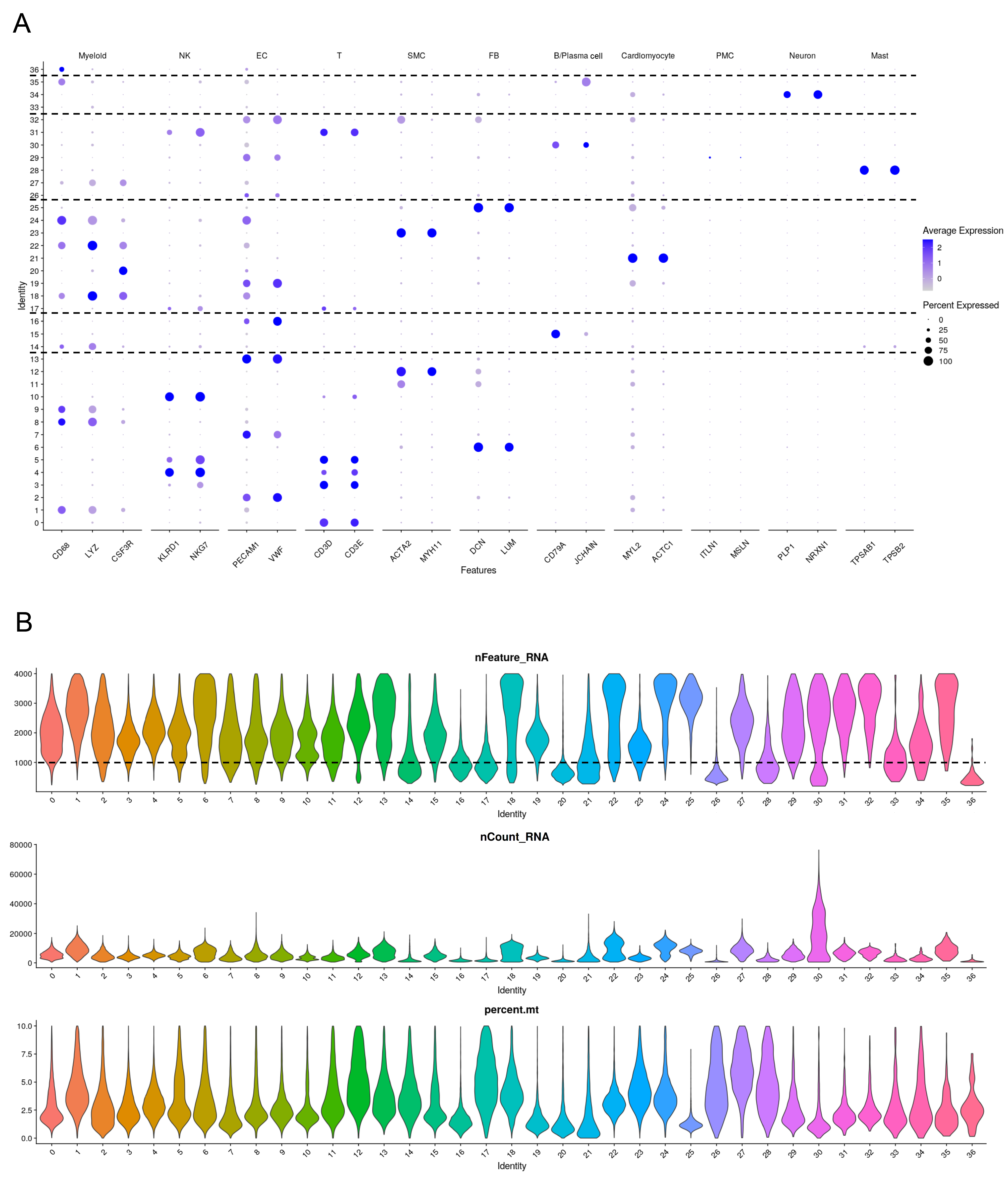


**Fig. S2. Cell type identification. A**, Dot plot showing the classical markers of each of the 37 clusters. Dot size corresponds to proportion of cells within the group expressing each gene, and dot color corresponds to its expression level. **B,** Count_RNA number, Feature_RNA number, mitochondrial RNA percentage of cells in 37 clusters. C32, C33, C35 and C36 were excluded because they contained doublets (C32 and C35) or low-quality cells (characterize low nubmer of features and unclear marker gene expression, C33 and C36). Thus, 247,684 cells were included for further analysis. NK, natural killer; EC, endothelial cell; PMC, pericardial mesothelial cell; SMC, smooth muscle cell; FB, fibroblast.


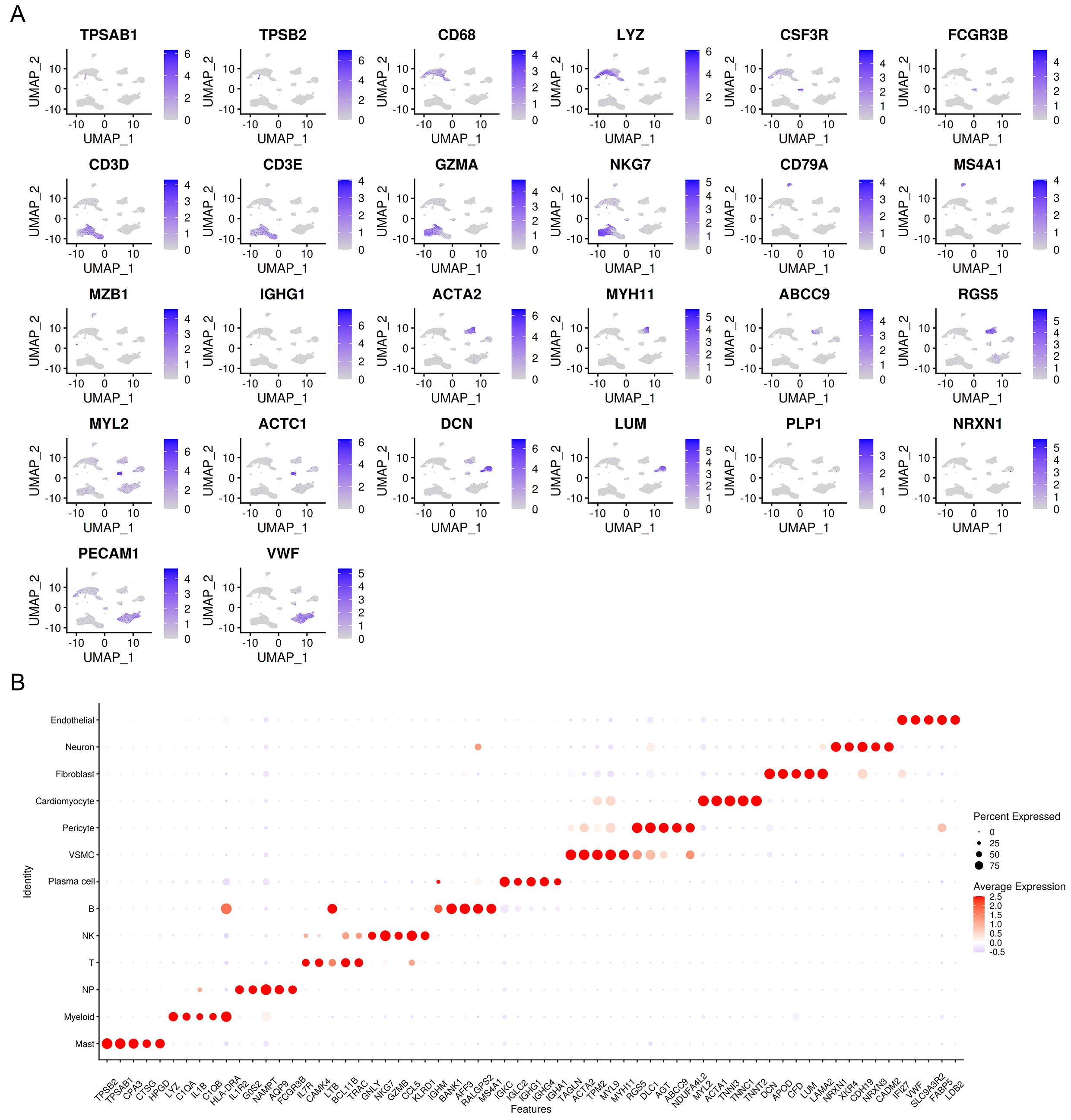


**Fig. S3. Cell type confirmation. A**, UMAP plot of classical markers in all cells. **B,** Dot plot showing the top 5 markers of each cell type. Dot size corresponds to proportion of cells within the group expressing each gene, and dot color corresponds to its expression level. UMAP, uniform manifold approximation and projection; NK, natural killer; EC, endothelial cell; PMC, pericardial mesothelial cell; SMC, smooth muscle cell; FB, fibroblast.


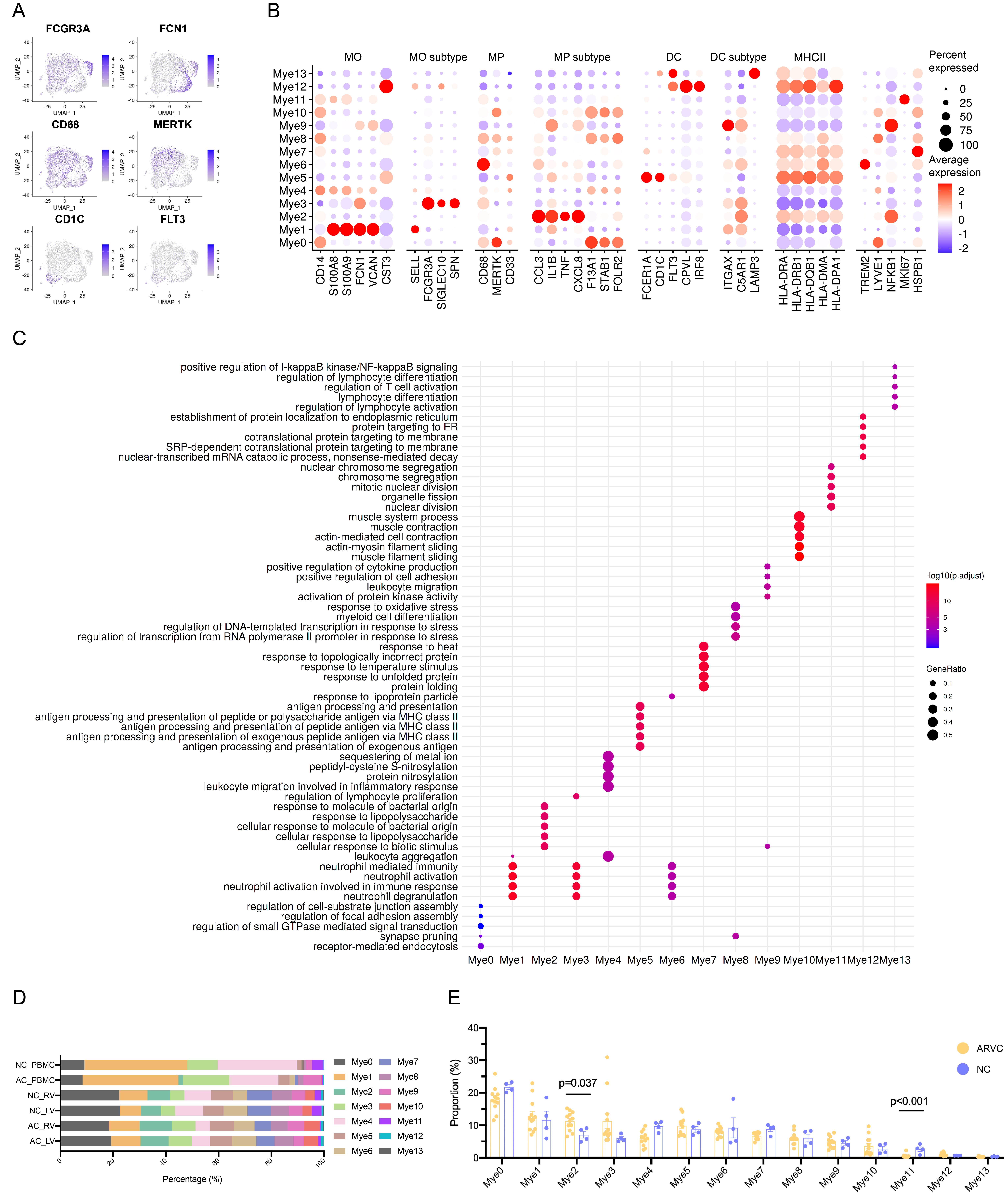


**Fig. S4. Myeloid subpopulations. A,** UMAP plot of classical markers in myeloid cells. **B,** Dot plot of classical markers and intersted gene in myeloid clusters. **C,** The top five enriched GOBP of each myeloid cluster. **D,** Phase and topographic region distribution of each cluster. **E**, Phase distribution of each cluster in ventricles. The Student’s t-test with Welch’s correction was performed to compare the log-transformed proportion of cell subpopulations between each pair of groups. For differential cellular proportion, p values were adjusted for multiple hypothesis testing using the Benjamini-Hochberg method. UMAP, uniform manifold approximation and projection; ARVC, arrhythmogenic right ventricular cardiomyopathy; NC, normal control; AC_LV, ARVC left ventricle; AC_RV, ARVC right ventricle; AC_PBMC, ARVC PBMC; NC_LV, NC left ventricle; NC_RV, NC right ventricle; PBMC peripherial blood mononuclear cell; GOBP, gene ontology biological process. Gene names mentioned in the main text were color-coded.


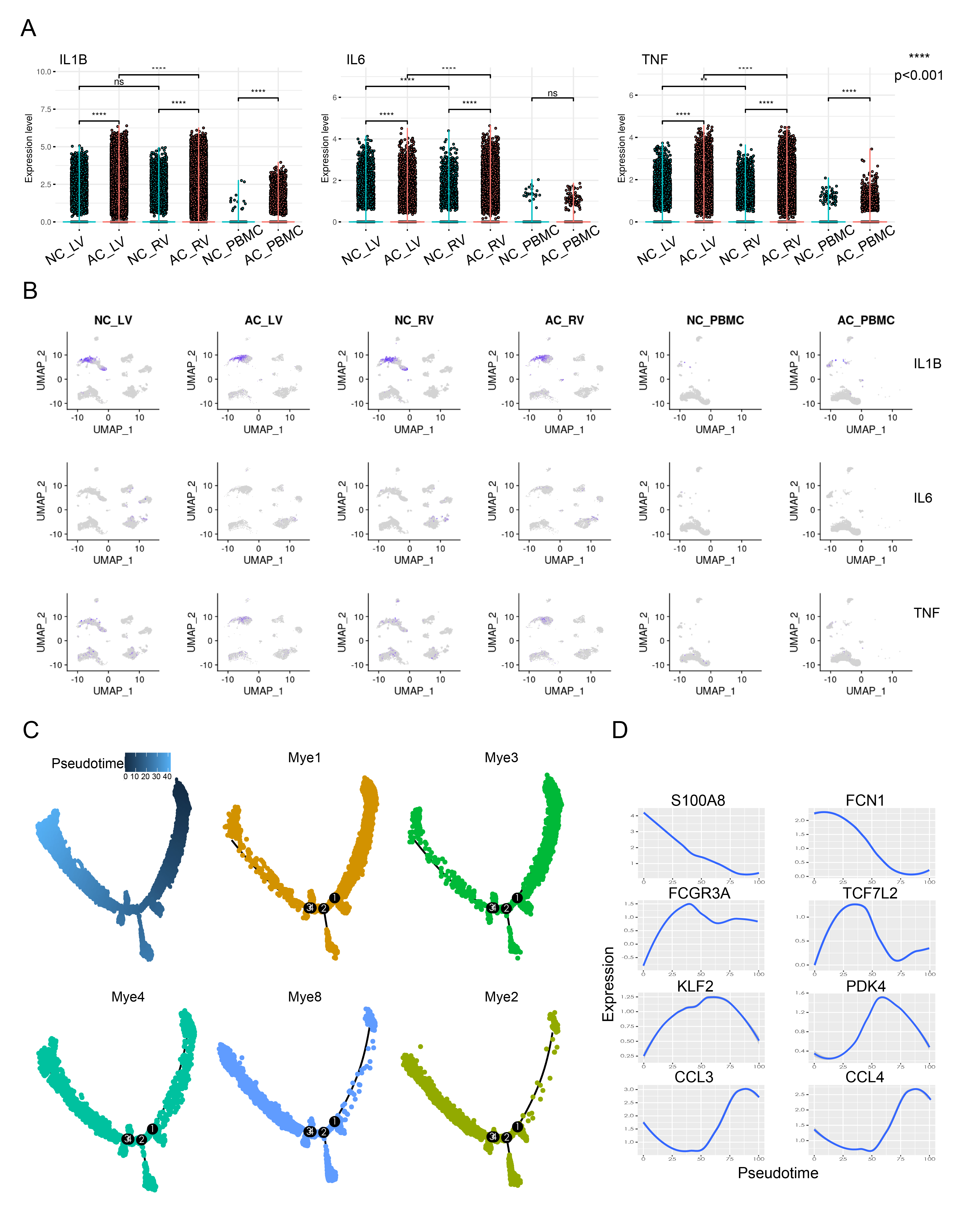


**Fig. S5. The expression of inflammatory genes and trajectory analysis of myeloid. A,** Violin plot of the expression of *IL1B*, *IL6*, *TNF* between different groups. **B**, UMAP plot of the three inflammatory genes in all cells. **C**, Trajectory analysis of myeloid clusters using monocle2. **D**, The expression of selected genes among Mye2 lineage using Slingshot. UMAP, uniform manifold approximation and projection; ARVC, arrhythmogenic right ventricular cardiomyopathy; NC, normal control; AC_LV, ARVC left ventricle; AC_RV, ARVC right ventricle; AC_PBMC, ARVC PBMC; NC_LV, NC left ventricle; NC_RV, NC right ventricle; PBMC peripherial blood mononuclear cell.

**Fig. S6. Fibroblast subpopulations. A,** Heatmap showing the top 50 DEGs of each fibroblast subcluster. **B**, Phase distribution of each cluster in ventricles. The Student’s t-test with Welch’s correction was performed to compare the log-transformed proportion of cell subpopulations between each pair of groups. For differential cellular proportion, p values were adjusted for multiple hypothesis testing using the Benjamini-Hochberg method. **C,** Percentage change tendency of each cluster among different phases and topographic regions and the contribution of each cell cluster to the different phases and topographic regions. **D**, Density plots reflecting the number of FB-cells along the FB6 (left) or FB5 (right) lineages stratified for different phases and topograhic. **E**, Profiling of marker genes along FB5 lineage. **F**, Immunofluorescence staining (left) and quantification (right) of ARVC and NC hearts for FB5; scale bar indicates 100μm**.** Each spot represents a sample. Mann–Whitney U test was performed to compare the cellular ratio of FB5 between two groups. ARVC, arrhythmogenic right ventricular cardiomyopathy; NC, normal control; AC_RV, ARVC right ventricle; AC_LV, ARVC left ventricle; NC_LV, NC left ventricle; NC_RV, NC right ventricle. Gene names mentioned in the main text were color-coded.


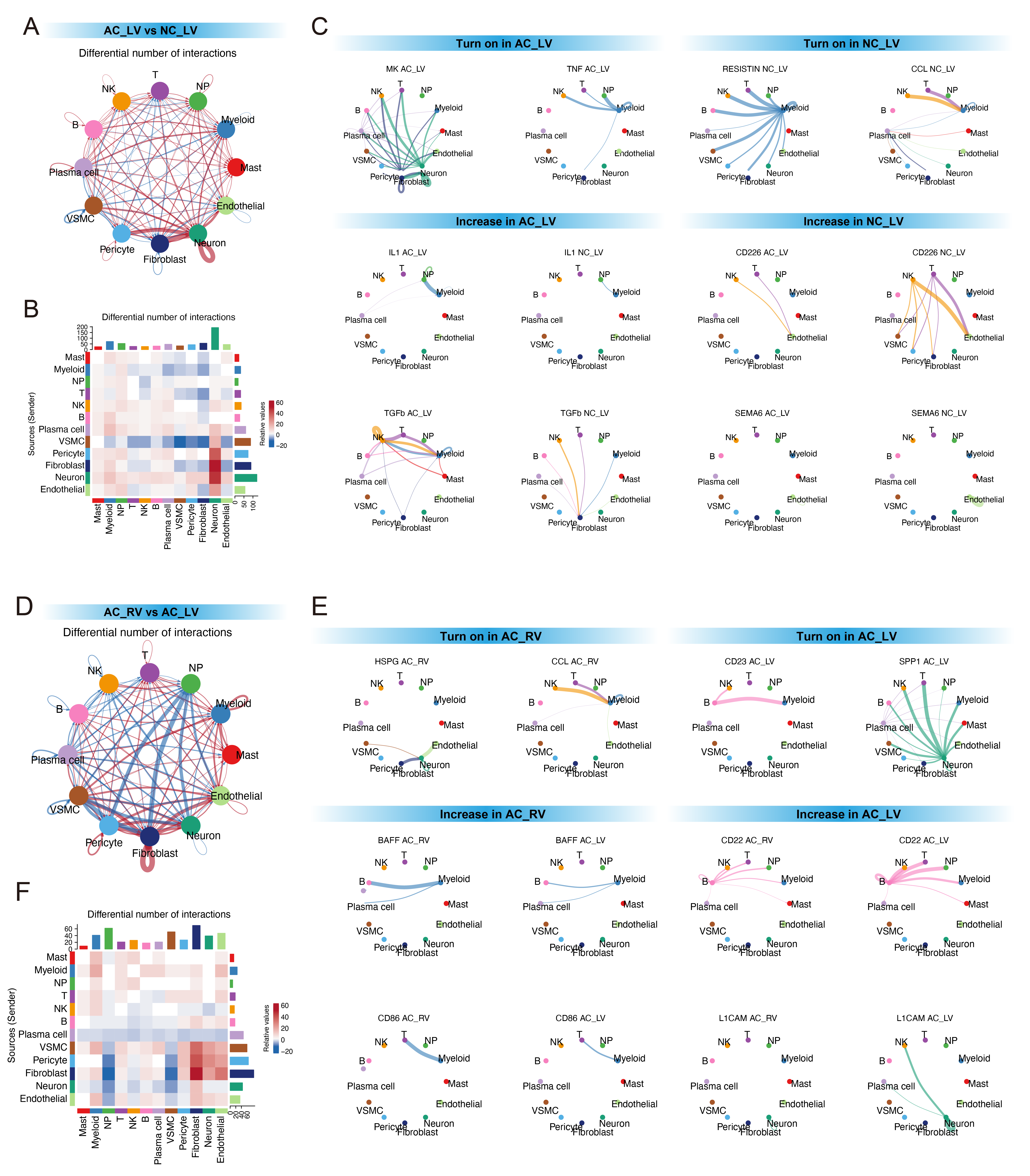


**Fig. S7. Predicted and altered cell-cell interactions in ARVC patient hearts. A, D,** Net plot showing the interaction number and weight among the 12 major cell clusters in the comparison of AC_RV and NC_RV (A) or AC_RV and AC_LV (D). Each dot indicates one cell cluster and its size is proportional to the number of cells in the cluster. The thickness of the lines connecting cell clusters indicates the differential interaction number (blue line indicates an increase in NC_RV, red line indicates an increase in AC_RV). **B, F**, Differential number of the cellular interactions between NC_RV and AC_RV (B) or AC_LV and AC_RV (F). Red and blue represent enrichment in the later one and the previous one, respectively. **C, E**, Circos plots showing the inferred intercellular communication network among the major cell types. ARVC, arrhythmogenic right ventricular cardiomyopathy; NC, normal control; AC_RV, ARVC right ventricle; AC_LV, ARVC left ventricle; NC_LV, NC left ventricle; NC_RV, NC right ventricle; NK, natural killer; NP, neutrophils; VSMC, vascular smooth muscle cell.


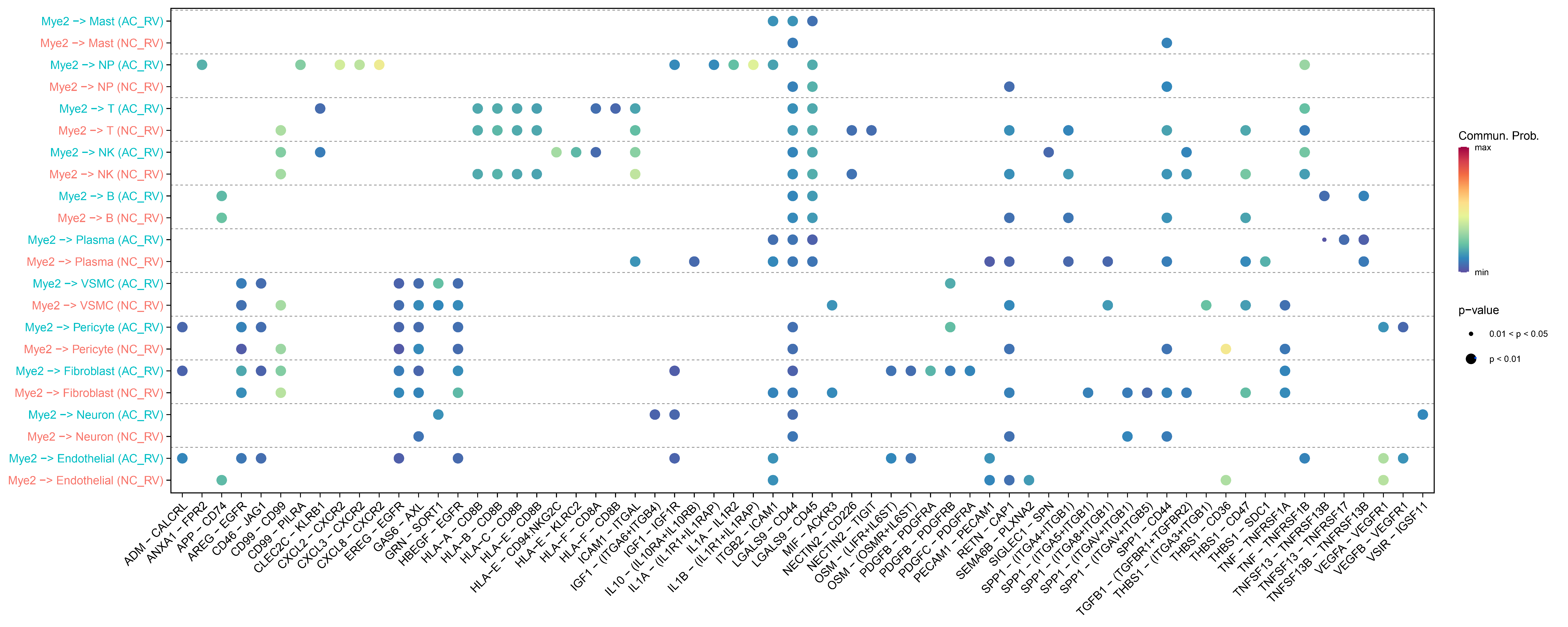


**Fig. S8. Predicted and altered Mye2-cell interactions in ARVC patient hearts.** Bubble plot showing the selected ligand–receptor interactions between Mye2 (ligand) to major cell types. ARVC, arrhythmogenic right ventricular cardiomyopathy; NC, normal control; AC_LV, ARVC left ventricle; AC_RV, ARVC right ventricle; NC_LV, NC left ventricle; NC_RV, NC right ventricle; NK, natural killer; NP, neutrophils; VSMC, vascular smooth muscle cell.


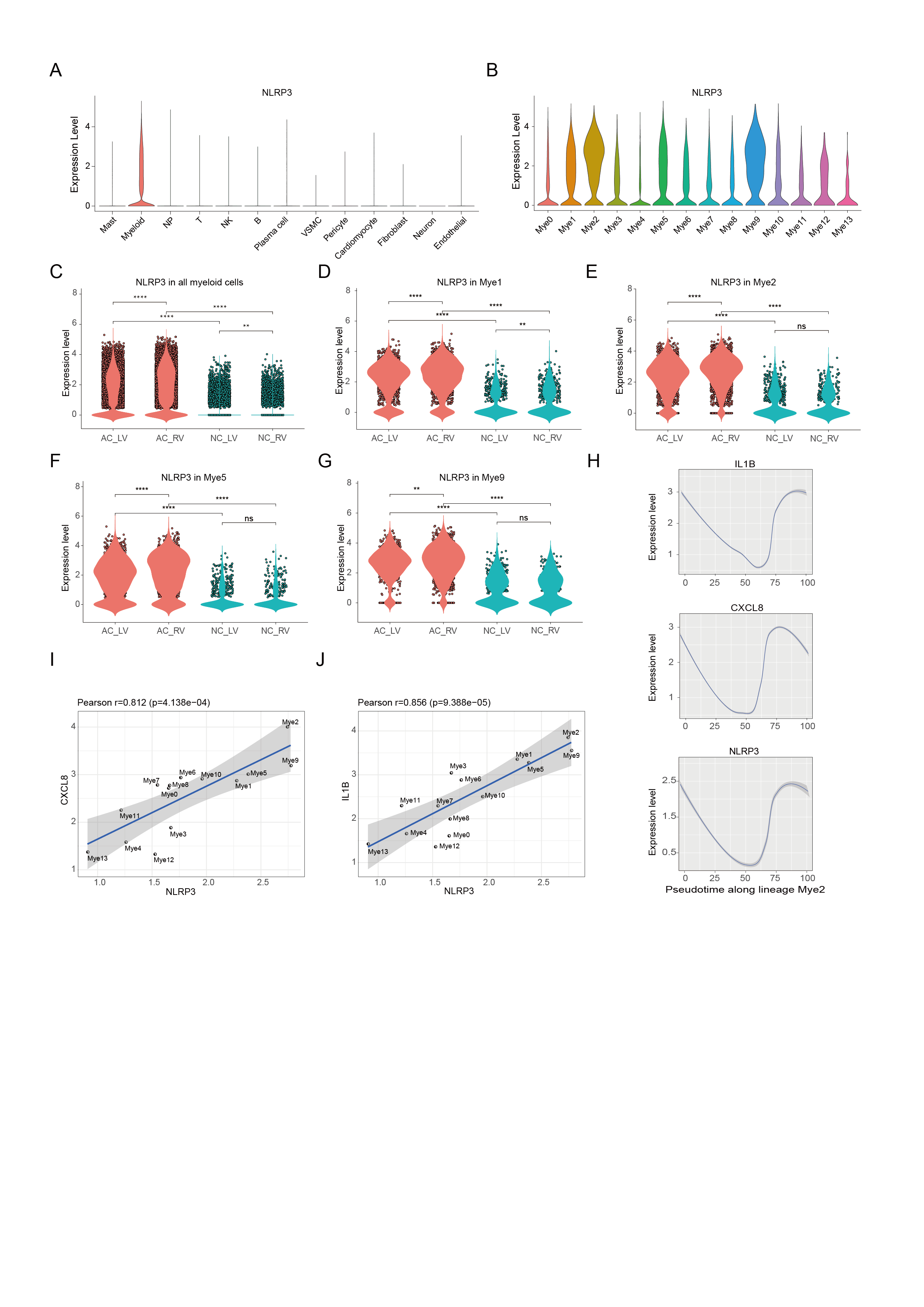


**Fig. S9. NLRP3 in ARVC patient hearts. A-B,** Violin plots showing the expression of *NLRP3* in myeloid celltypes (**A**) or myeloid cells among different phases and topographic regions (**B**). **C,** The expression of *IL1B*, *CXCL8* and *NLRP3* along Mye2 lineage. **D,** Correlations between the *NLRP3* expression level and the inflammatory genes of each myeloid cell cluster. ARVC, arrhythmogenic right ventricular cardiomyopathy; NC, normal control; AC_LV, ARVC left ventricle; AC_RV, ARVC right ventricle; NC_LV, NC left ventricle; NC_RV, NC right ventricle.


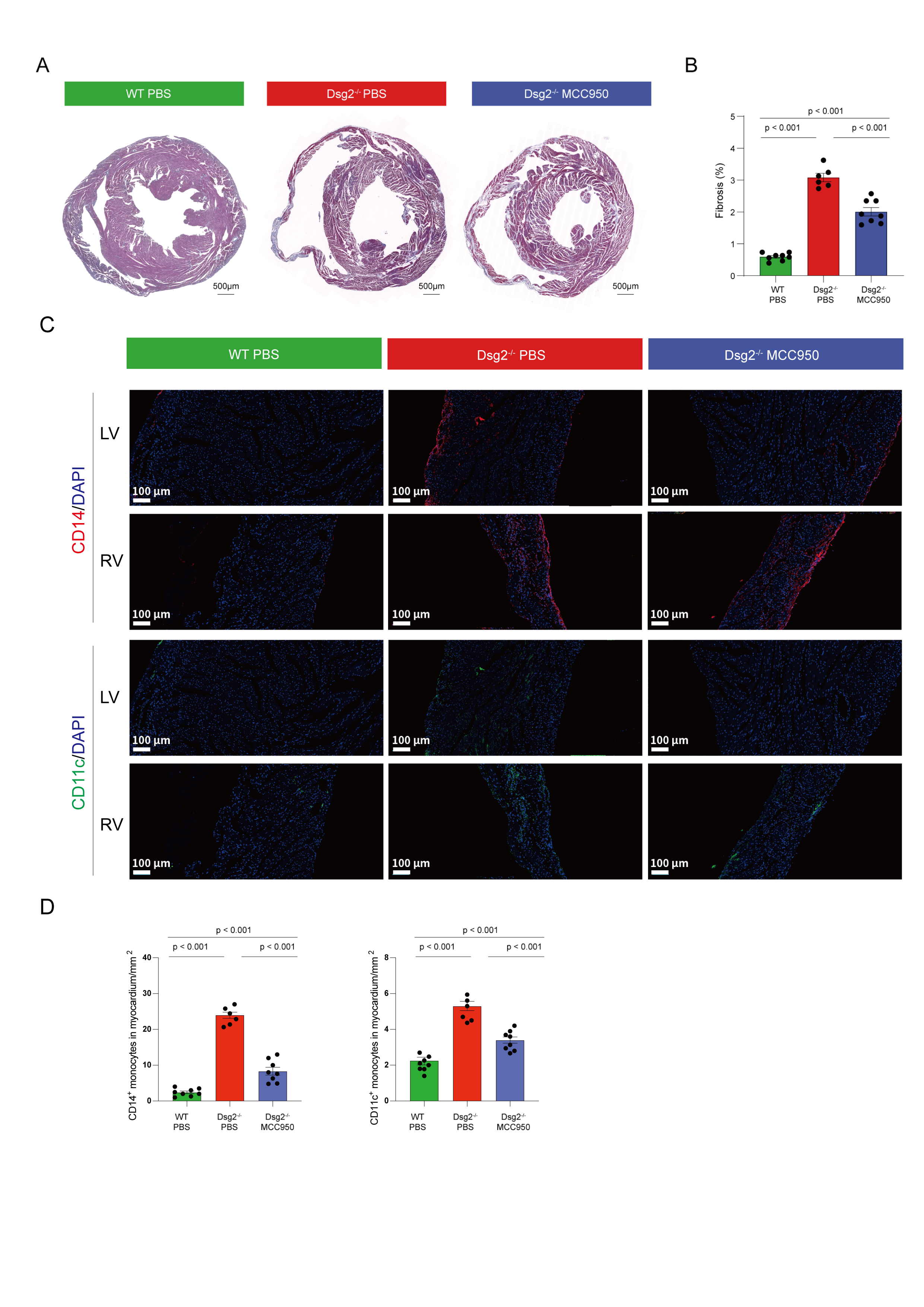


**Fig. S10.** **Pharmacological inhibition of NLRP3 significantly alleviate the fibrosis and inflammation in ARVC mouse. A,** Representative gross pathology of the hearts stained with Masson staining from PBS infused wild type and Dsg2^mut/mut^ mice, and MCC950 infused Dsg2^mut/mut^ mice at 12 weeks of age. Scale bar, 500 µm. **B,** Quantification of the percent of cardiac fibrosis in **A**. Each spot represents one sample. Data are mean ± SD. Mann–Whitney U test was performed to compare the percentage of cardiac fibrosis. **C,** Multiple labeling staining for CD11c^+^ DC cells (green) and CD14^+^ monocytes (red); scale bar indicates 100μm**. D,** Quantification of the cell ratio in **C**. Each spot represents one sample. Data are mean ± SD. Mann–Whitney U test was performed to compare the cellular ratio of DC cells and monocytes. LV, left ventricle; RV, right ventricle.
